# Supplementary material for: Estimating repeat spectra and genome length from low-coverage genome skims with RESPECT
Source: PLoS Comput Biol. 2021 Nov 15;17(11):e1009449. doi: 10.1371/journal.pcbi.1009449 (PMC8629397; doi:10.1371/journal.pcbi.1009449)
Supplement: S1 Appendix — Detailed mathematical derivations and supplementary tables. Table A: SRA preprocessing results. Table B: List of species with recent WGD events. (PDF) [file pcbi.1009449.s001.pdf]

Supplementary methods

Initial estimate of parameters. With the assumption that a genome has no repeating k-mers $\mathbf{r} = [L, 0, 0, \ldots]$, Eq. (5) reduces to

$$\mathbb{E}\left[o_h\right] = m_h = \begin{cases} L\lambda(1-\epsilon)^k e^{-\lambda(1-\epsilon)^k} + L\lambda(1-(1-\epsilon)^k) & h = 1 \\ L\frac{(\lambda(1-\epsilon)^k)^h}{h!} e^{-\lambda(1-\epsilon)^k} & h > 1 \end{cases} . \tag{1}$$

We use the method of moments (see e.g., Section 7.6 of[1]) and set $m_h = o_h$ to estimate the underlying parameters λ and ϵ. Specifically, let $h^* = \arg\max_{h>1} o_h$ be the multiplicity with the largest number of observed k-mers (excluding the unique ones $h = 1$). We use o_{h^*+1}/o_{h^*} to estimate $\lambda_{\text{ef}} = \lambda(1-\epsilon)^k$

$$\lambda_{\text{ef}} = \frac{(h^*+1)o_{h^*+1}}{o_{h^*}} . \tag{2}$$

Then, using o_{h^*}/o_1, we estimate λ as

$$\lambda = \lambda_{\text{ef}}^h e^{-\lambda_{\text{ef}}} \frac{o_1}{h^*!\, o_{h^*}} - \lambda_{\text{ef}} e^{-\lambda_{\text{ef}}} + \lambda_{\text{ef}} , \tag{3}$$

and estimate ϵ from the ratio of λ_{ef} and λ

$$\epsilon = 1 - (\frac{\lambda_{\text{ef}}}{\lambda})^{1/k} . \tag{4}$$

Least-squares estimate of repeat spectrum. Consider the cost function defined in Eq. (6) with $p = 2$ and $w_h = 1$ for all h

$$\mathcal{E}_{\mathbf{w},p}(\mathbf{P}, \mathbf{r}, \epsilon, \mathbf{o}) = \left(\sum_h \left|m_h - o_h\right|^2\right)^{1/2} = \left(\sum_h \left|(\mathbf{r}\mathbf{P}^{\mathbf{T}} + \mathbf{1}_{h=1}E)_h - o_h\right|^2\right)^{1/2} . \tag{5}$$

We considered the simplest sequencing-error-free case ($\epsilon = 0$), where coverage λ was known. Therefore, $\mathbb{E}\left[\mathbf{O}\right] = \mathbf{m} = \mathbf{r}\mathbf{P}^{\mathbf{T}}$, where $\mathbf{P}$ is an $n \times n$ matrix with

$$P_{hj} = e^{-j\lambda} \frac{(j\lambda)^h}{h!}. \tag{6}$$

$\mathbf{P}$ can be decomposed as $\mathbf{P} = \mathbf{\Lambda}\mathbf{V}\mathbf{E}$ where $\mathbf{\Lambda}$ is a diagonal matrix with $\mathbf{\Lambda}_{hh} = \frac{\lambda^h}{h!}$, $\mathbf{E}$ is a diagonal matrix with $\mathbf{E}_{jj} = je^{-j\lambda}$, and $\mathbf{V}$ is the transpose of a Vandermonde matrix with the second column given by the vector $(1, 2, 3, \ldots, n)^T$; thus $V_{hj} = j^{h-1}$, and

$$\mathbf{V} = \begin{bmatrix} 1 & 1 & 1 & \cdots & 1 \\ 1 & 2 & 3 & \cdots & n \\ 1 & 2^2 & 3^2 & \cdots & n^2 \\ \vdots & \vdots & \vdots & \ddots & \vdots \\ 1 & 2^{n-1} & 3^{n-1} & \cdots & n^{n-1} \end{bmatrix}. \tag{7}$$

Note that $\mathbf{\Lambda}$ and $\mathbf{E}$ are diagonal matrices with non-zero diagonal elements and hence, they are non-singular. Also, since V is a Vandermonde matrix, we have $\det(\mathbf{V}) = \prod_{1\le i<j\le n}(j-i) > 0$ which renders $\mathbf{V}$ a non-singular matrix. Thus, it enables us to use the estimate $\mathbf{r}^{(\text{est})} = \mathbf{o}\mathbf{P}^{-\mathbf{T}}$. However, for the case $\lambda = 1$ we prove that

$$\text{cond}(\mathbf{P}) \ge c\frac{2^n}{n},$$

suggesting a highly ill-conditioned matrix, and making the LS estimates unreliable.

Bound on $\text{cond}(\mathbf{P})$**.** To establish the aforementioned bound for $\text{cond}(\mathbf{P})$, as $\mathbf{P}$ is non-singular, we have (see e.g., Section 3.8 of[2])

$$\text{cond}(\mathbf{P}) = \|\mathbf{P}\|\|\mathbf{P}^{-1}\| = \|\mathbf{\Lambda}\mathbf{V}\mathbf{E}\| \cdot \|\mathbf{E}^{-1}\mathbf{V}^{-1}\mathbf{\Lambda}^{-1}\|, \tag{8}$$

where $\|\mathbf{A}\|$ is the induced 2-norm of the matrix $\mathbf{A}$. Since both $\mathbf{E}$ and $\mathbf{\Lambda}$ are diagonal, we have

$$\begin{aligned} \text{cond}(\mathbf{\Lambda}) = \text{cond}(\mathbf{\Lambda}^{-1}) &= \frac{\max_i \left\{\lambda^i/i!\right\}}{\min_i \left\{\lambda^i/i!\right\}} = \frac{\lambda^{\lfloor\lambda\rfloor}/\lfloor\lambda\rfloor!}{\min(1, \lambda^n/n!)} \\ \text{cond}(\mathbf{E}) = \text{cond}(\mathbf{E}^{-1}) &= \frac{\max_i \left\{ie^{-i\lambda}\right\}}{\min_i \left\{ie^{-i\lambda}\right\}} = \frac{f(\lambda)}{\min(e^{-\lambda}, ne^{-n\lambda})}, \end{aligned}$$

where

$$f(\lambda) = \begin{cases} \max(\lfloor\lambda\rfloor e^{-\lambda\lfloor\lambda\rfloor}, (\lfloor\lambda\rfloor+1)e^{-\lambda(\lfloor\lambda\rfloor+1)}) & \lambda < 1 \\ e^{-\lambda} & \lambda \ge 1 \end{cases}.$$

For the simplicity of exposition, we use $\lambda = 1$. Hence,

$$\begin{aligned} \text{cond}(\mathbf{\Lambda}) &= \text{cond}(\mathbf{\Lambda}^{-1}) = n!, \\ \text{cond}(\mathbf{E}) &= \text{cond}(\mathbf{E}^{-1}) = \frac{1}{n}e^{n-1}. \end{aligned} \tag{9}$$

Note that for the 2-norm $\|\cdot\|$, we have

$$\|\mathbf{AB}\| \leq \|\mathbf{A}\|\|\mathbf{B}\| \tag{10}$$

hence, for any matrix $\mathbf{B}$ and any invertible matrix $\mathbf{A}$, we have $\|\mathbf{B}\| = \|\mathbf{A}^{-1}\mathbf{AB}\| \leq \|\mathbf{A}^{-1}\|\|\mathbf{AB}\|$, implying

$$\|\mathbf{AB}\| \geq \frac{\|\mathbf{B}\|}{\|\mathbf{A}^{-1}\|}. \tag{11}$$

By repeated application of this inequality to Eq. (8) and using (9), we have

$$\operatorname{cond}(\mathbf{P}) \geq \frac{\operatorname{cond}(\mathbf{V})}{\operatorname{cond}(\mathbf{E}) \cdot \operatorname{cond}(\mathbf{\Lambda})} = \frac{n}{n!e^{n-1}} \operatorname{cond}(\mathbf{V}). \tag{12}$$

To show that $\operatorname{cond}(\mathbf{P})$ grows exponentially, we use the following lemma.

Lemma 1 *For the matrix* $\mathbf{V}$ *given in* (7)*, we have*

$$c\frac{1}{n^{3/2}}(2n)^n \leq \operatorname{cond}(\mathbf{V}) \leq C(2n)^n. \tag{13}$$

for some $c, C > 0$*.*

Proof. Since $\mathbf{V}$ is non-singular, $\operatorname{cond}(\mathbf{V}) = \|\mathbf{V}\|\|\mathbf{V}^{-1}\|$ and hence, it remains to bound $\|\mathbf{V}\|$ and $\|\mathbf{V}^{-1}\|$. To do this, we use the following inequalities relating several norms of a matrix $\mathbf{A}$ (see e.g., Section 10.4.4 of[3])

$$\frac{1}{\sqrt{n}}\|\mathbf{A}\|_F \leq \|\mathbf{A}\| \leq \|\mathbf{A}\|_F, \tag{14}$$

$$\frac{1}{\sqrt{n}}\|\mathbf{A}\|_\infty \leq \|\mathbf{A}\| \leq \sqrt{n}\|\mathbf{A}\|_\infty, \tag{15}$$

where $\|\mathbf{A}\|_F$ and $\|\mathbf{A}\|_\infty$ are the Frobenius and the induced ∞-norm of $\mathbf{A}$, respectively.

1. **Bounding** $\|\mathbf{V}\|$: Using (14) and $\|\mathbf{V}\|_F^2 = \sum_{i=1}^n \sum_{j=1}^n j^{2i-2} \geq n^{2(n-1)}$, we get

   $$\|\mathbf{V}\|_F \geq n^{n-1}.$$

   For an upper bound on $\|\mathbf{V}\|_F$, we have

$$
\begin{aligned}
\|\mathbf{V}\|_F^2 &= \sum_{i=1}^{n}\sum_{j=1}^{n} j^{2i-2} \\
&\leq \sum_{i=1}^{n}\left(n^{2i-2} + \int_{1}^{n} x^{2i-2}dx\right) \\
&= \sum_{i=1}^{n}\left(n^{2i-2} + \frac{n^{2i-1}-1}{2i-1}\right) \\
&= \sum_{i=1}^{n} n^{2i-2} + \sum_{i=1}^{n}\frac{n^{2i-1}}{2i-1},
\end{aligned}
$$

where the inequality follows from x^k being a monotonically increasing function for positive x and all $k \geq 1$. But

$$
\sum_{i=1}^{n} n^{2i-2} = \sum_{i=1}^{n}(n^2)^{i-1} = \frac{n^{2n}-1}{n^2-1} \leq \frac{n^{2n}}{n^2-1}. \tag{16}
$$

Therefore, for $n \geq 3$, we have

$$
\begin{aligned}
\|\mathbf{V}\|_F^2 &\leq \frac{n^{2n}}{n^2-1} + \sum_{i=1}^{n}\frac{n^{2i-1}}{2i-1} \\
&= \frac{n^{2n}}{n^2-1} + \frac{n^{2n-1}}{2n-1} + \sum_{i=1}^{n-1}\frac{n^{2i-1}}{2i-1} \\
&\leq \frac{n^{2n}}{n^2-1} + \frac{n^{2n-1}}{2n-1} + (n-1)\frac{n^{2n-3}}{2(n-1)-1} \\
&\leq (\frac{1}{1-n^{-2}} + \frac{1}{2-n^{-2}} + \frac{1}{2n-3})n^{2n-2} \\
&\leq 2n^{2n-2},
\end{aligned}
$$

where the second inequality follows from $\frac{n^{2i-1}}{2i-1}$ being an increasing function of i for $n \geq 3$. It can be verified that the above bound holds for $n = 1, 2$ too. Using this fact, and the lower bound on $\|\mathbf{V}\|_F$, we have

$$
\frac{1}{\sqrt{n}} n^{n-1} \leq \|\mathbf{V}\| \leq \sqrt{2} n^{n-1}. \tag{17}
$$

2. **Bounding** $\|\mathbf{V}^{-1}\|$: Using Theorem 1 by Gautschi (1962)[4], we have:

$$
\|\mathbf{V}^{-1}\|_\infty = \max_i \prod_{j\neq i} \frac{1+j}{|j-i|}. \tag{18}
$$

Note that for a fixed i,

$$\prod_{j\neq i}(1+j) = \frac{(n+1)!}{i+1},$$

and

$$\prod_{j\neq i}\frac{1}{|j-i|} = \frac{1}{(i-1)!(n-i)!}.$$

Therefore,

$$\prod_{j\neq i}\frac{1+j}{|j-i|} = \frac{(n+1)!}{(i+1)(i-1)!(n-i)!} = \binom{n}{i}\frac{i}{i+1}(n+1).$$

Replacing this in (18), we get

$$\frac{1}{2}(n+1)\max_i\binom{n}{i} \leq \|\mathbf{V}^{-1}\|_\infty \leq (n+1)\max_i\binom{n}{i}. \tag{19}$$

But $\max_i\binom{n}{i} = \binom{n}{\lfloor n/2\rfloor}$ and hence, by the Sterling's approximation[5], we have

$$a\frac{1}{\sqrt{n}}2^n \leq \max_i\binom{n}{i} \leq A\frac{1}{\sqrt{n}}2^n$$

for some $a < A$. Using this in (19), we arrive at

$$b\sqrt{n}2^n \leq \|\mathbf{V}^{-1}\|_\infty \leq B\sqrt{n}2^n,$$

for some $b, B > 0$. Therefore, using (15), we get

$$b2^n \leq \|\mathbf{V}^{-1}\| \leq Bn2^n. \tag{20}$$

Finally, combining the bounds (17) and (20) on $\|\mathbf{V}\|$ and $\|\mathbf{V}^{-1}\|$, respectively, and using $\text{cond}(\mathbf{V}) = \|\mathbf{V}\|\|\mathbf{V}^{-1}\|$, we get the desired result

$$c\frac{1}{n^{3/2}}(2n)^n \leq \text{cond}(\mathbf{V}) \leq C(2n)^n,$$

for some constants $c, C > 0$.

■

Now, we are ready to show that $\mathbf{P}$ is a highly ill-conditioned matrix.

Lemma 2 *For the matrix* $\mathbf{P}$ *(given by* (6)*), we have*

$$\text{cond}(\mathbf{P}) \geq c\frac{2^n}{n}, \tag{21}$$

for some $c > 0$.

Proof. By the application of the lower-bound of Lemma 1 to (12), we have

$$\mathrm{cond}(\mathbf{P}) \geq \frac{n}{n!e^{n-1}}\,\mathrm{cond}(\mathbf{V}) \geq \frac{n}{n!e^{n-1}} \cdot c\frac{1}{n^{3/2}}(2n)^n = \frac{c(2n)^n}{\sqrt{n}n!e^{n-1}}.$$

Therefore, using the Sterling's approximation $n! \leq n^{n+1/2}e^{-(n-1)}$, we get

$$\mathrm{cond}(\mathbf{P}) \geq c\,\frac{2^n}{n}$$

for some constant $c > 0$. ■

Since the bound (21) can be written as

$$\mathrm{cond}(\mathbf{P}) \geq c\,2^{n-\log_2 n},$$

and for any $\epsilon > 0$, we have $n - \log_2 n \geq (1-\epsilon)n$ for sufficiently large n, we have $\mathrm{cond}(\mathbf{P}) \geq c2^{(1-\epsilon)n}$. Therefore, $\mathrm{cond}(\mathbf{P})$ grows exponentially and in fact, $\mathrm{cond}(\mathbf{P}) = \Omega(\alpha^n)$ for any $\alpha < 2$.

References

[1] DeGroot MH, Schervish MJ. Probability and statistics. Pearson Education; 2012.

[2] Meyer CD. Matrix analysis and applied linear algebra. vol. 71. Siam; 2000.

[3] Petersen KB, Pedersen MS. The Matrix Cookbook; 2012. http://www2.compute.dtu.dk/pubdb/pubs/3274-full.html.

[4] Gautschi W. On inverses of Vandermonde and confluent Vandermonde matrices. Numerische Mathematik. 1962;4(1):117–123. doi:10.1007/BF01386302.

[5] Robbins H. A remark on Stirling's formula. The American mathematical monthly. 1955;62(1):26–29.

Supplementary tables

**Species**	Common name	Run accession	Genome length (RefSeq)	Duplication	Contamination	Total	RESPECT error	CovEst error
Pelecanus crispus	Dalmatian pelican	SRR959397	1160924693	4%	3%	7%	-4%	-42%
Zea mays	Maize	SRR2960981	2135083061	1%	7%	8%	-8%	-90%
Leptosomus discolor	Cuckoo roller	SRR956935	1136244952	6%	3%	9%	4%	-17%
Merops nubicus	Northern carmine bee-eater	SRR958515	1062961556	6%	3%	9%	4%	-13%
Nicotiana tabacum	Cultivated tobacco	SRR955758	3643471356	7%	3%	10%	3%	-85%
Aegilops tauschii	Tausch's goatgrass	SRR5170323	4327321625	5%	7%	12%	-11%	-91%
Frankliniella occidentalis	Western flower thrips	SRR1300141	274989634	7%	5%	12%	0%	-2%
Mustela putorius	Domestic ferret	SRR085103	2410879678	9%	4%	13%	-60%	-61%
Salmo salar	Atlantic salmon	SRR1264544	2966890203	7%	8%	15%	-5%	-89%
Ceratitis capitata	Mediterranean fruit fly	SRR847379	436490799	10%	7%	17%	-3%	-40%
Athene cunicularia	Burrowing owl	SRR6670174	1157069330	15%	4%	19%	-5%	-6%
Manis javanica	Sunda pangolin	SRR3929782	2547395906	7%	16%	23%	-2%	-58%
Elephantulus edwardii	Cape sengi	SRR387354	3843982861	9%	15%	24%	18%	-90%
Propithecus coquereli	Coquerel's sifaka	SRR1657020	2798152141	7%	20%	27%	-41%	-54%
Perca flavescens	Yellow perch	SRR8482300	877456336	1%	28%	29%	3%	-69%
Fopius arisanus	A Braconid wasp	SRR1560668	153631861	28%	2%	30%	-2%	-43%
Sturnus vulgaris	Common starling	SRR2240710	1036755994	15%	20%	35%	7%	-44%
Charadrius vociferus	Killdeer	SRR944000	1219859583	37%	1%	38%	-3%	-84%
Linepithema humile	Argentine ant	SRR059226	219500750	10%	29%	39%	-8%	-34%
*Cyprinodon variegatus	Sheepshead minnow	SRR1261820	1035184475	24%	19%	43%		
*Bombus impatiens	Eastern bumble bee	SRR1575221	246856484	12%	32%	44%		
*Priapulus caudatus	Penis worm	SRR649590	511738253	18%	27%	45%		
*Diaphorina citri	Citrus psylla	SRR189236	485705082	39%	11%	50%		
*Drosophila miranda	Fruit fly	SRR789694	136728780	49%	12%	61%		
*Musca domestica	Housefly	SRR650115	750403944	12%	50%	62%		
*Stegastes partitus	Bicolor damselfish	SRR649426	800491834	34%	41%	75%		
*Bos mutus	Wild yak	SRR361227	2645161911	64%	12%	76%		
*Populus euphratica	Desert poplar	SRR616245	496032534	94%	4%	98%		
*Carlito syrichta	Philippine tarsier	SRR3502922	3453864774	59%	82%	141%		

Table A: **SRA preprocessing results.** The percentage of identified duplicate and contaminant reads are provided. Samples with more than 40% total duplication plus contamination (marked with an asterisk) were discarded.

Table B: **List of species with recent WGD events.**

**Species**	**UR**	**HCRM**
Amborella trichopoda	0.85	48
Ananas comosus	0.71	113
Arabidopsis lyrata	0.71	26
Arabidopsis thaliana	0.91	14
Asparagus officinalis	0.51	60
Beta vulgaris	0.74	77
Brachypodium distachyon	0.83	5
Brassica napus	0.40	25
Brassica oleracea	0.71	21
Brassica rapa	0.80	112
Cajanus cajan	0.67	38
Cannabis sativa	0.51	67
Carica papaya	0.81	21
Chenopodium quinoa	0.50	165
Chlamydomonas reinhardtii	0.85	242
Chondrus crispus	0.51	6
Cicer arietinum	0.66	106
Citrus clementina	0.75	41
Citrus sinensis	0.68	154
Coccomyxa subellipsoidea	0.96	27
Cucumis melo	0.84	49
Cucumis sativus	0.82	26
Daucus carota	0.66	35
Dendrobium catenatum	0.79	30
Eucalyptus grandis	0.69	50
Fragaria vesca	0.82	57
Glycine max	0.65	107
Glycine soja	0.63	95
Gossypium raimondii	0.76	13
Ipomoea nil	0.55	547
Ipomoea triloba	0.67	193
Jatropha curcas	0.79	55
Juglans regia	0.60	45
Lactuca sativa	0.53	36
Lupinus angustifolius	0.73	60
Malus domestica	0.65	38

Continued on next page

Table B – *Continued from previous page*

**Species**	**UR**	**HCRM**
Manihot esculenta	0.67	17
Medicago truncatula	0.77	57
Morus notabilis	0.72	57
Musa acuminata	0.79	85
Nelumbo nucifera	0.82	27
Nicotiana attenuata	0.49	46
Nicotiana sylvestris	0.56	41
Nicotiana tabacum	0.57	20
Nicotiana tomentosiformis	0.60	37
Nymphaea colorata	0.78	152
Olea europaea	0.65	40
Oncorhynchus kisutch	0.52	370
Oncorhynchus mykiss	0.59	221
Oncorhynchus nerka	0.68	182
Oncorhynchus tshawytscha	0.57	92
Oryza sativa	0.75	44
Panicum hallii	0.56	28
Papaver somniferum	0.44	18
Phalaenopsis equestris	0.72	25
Phoenix dactylifera	0.77	21
Physcomitrella patens	0.73	47
Populus trichocarpa	0.77	49
Prunus avium	0.68	155
Prunus mume	0.77	60
Prunus persica	0.75	69
Punica granatum	0.72	42
Pyrus x bretschneideri	0.54	133
Quercus lobata	0.63	62
Quercus suber	0.62	60
Ricinus communis	0.73	69
Rosa chinensis	0.61	70
Salmo salar	0.48	356
Salmo trutta	0.55	330
Salvelinus alpinus	0.69	185
Selaginella moellendorffii	0.42	39
Sesamum indicum	0.84	12

Continued on next page

Table B – *Continued from previous page*

**Species**	**UR**	**HCRM**
Setaria italica	0.76	14
Solanum lycopersicum	0.80	39
Solanum pennellii	0.76	39
Solanum tuberosum	0.74	54
Sorghum bicolor	0.52	46
Syzygium oleosum	0.75	48
Theobroma cacao	0.79	14
Vitis vinifera	0.73	44
Volvox carteri	0.80	151
Zea mays	0.32	32
Ziziphus jujuba	0.58	90
