# Supplementary figures and images for: Estimating repeat spectra and genome length from low-coverage genome skims with RESPECT
Source: PLoS Comput Biol. 2021 Nov 15;17(11):e1009449. doi: 10.1371/journal.pcbi.1009449 (PMC8629397; doi:10.1371/journal.pcbi.1009449)

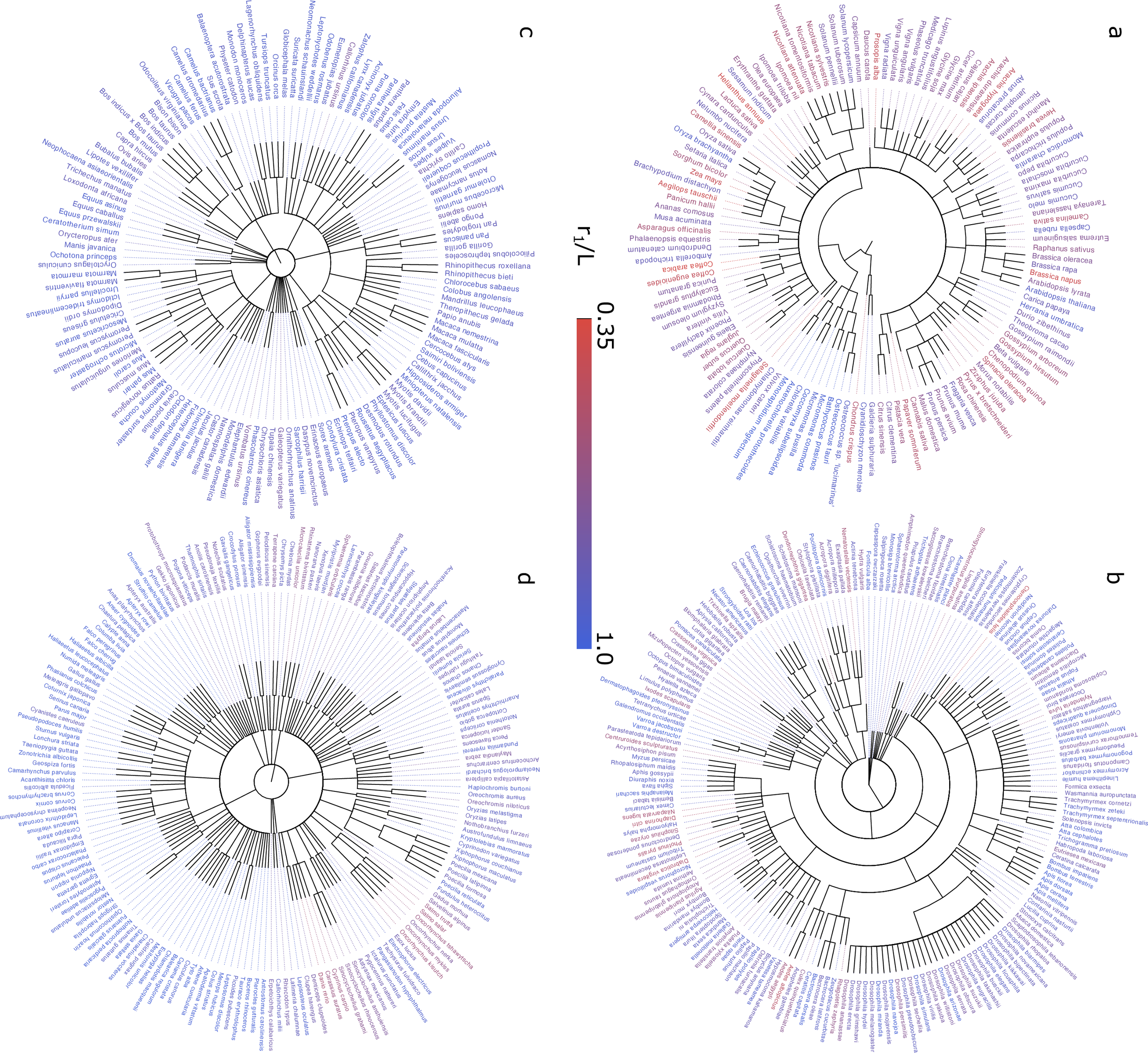

Supplement: S1 Fig — A: Plants, B: Invertebrates, C: Mammals, D: Other vertebrates. (TIF) [file pcbi.1009449.s002.tif]

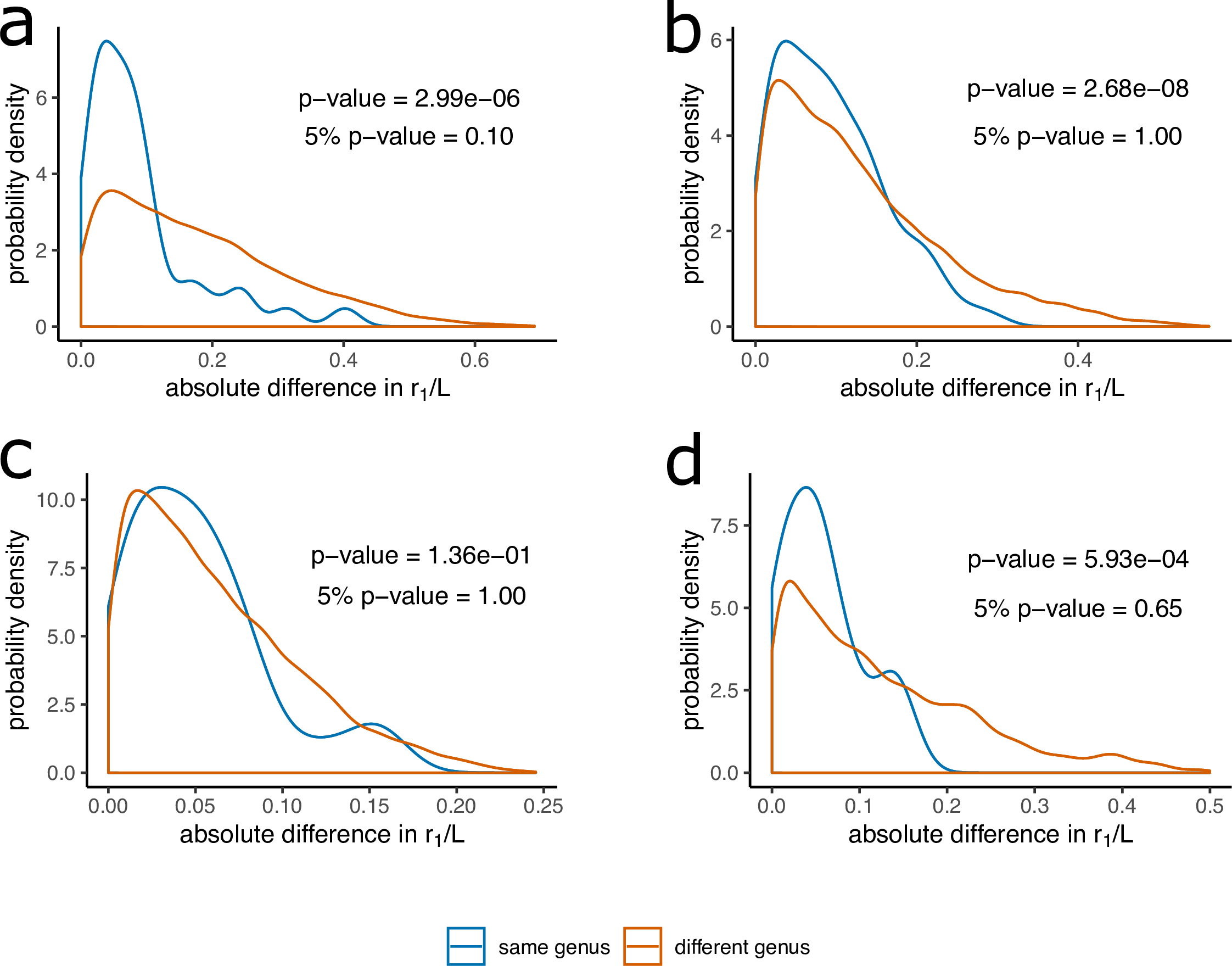

Supplement: S2 Fig — A: Plants, B: Invertebrates, C: Mammals, D: Other vertebrates. (TIF) [file pcbi.1009449.s003.tif]

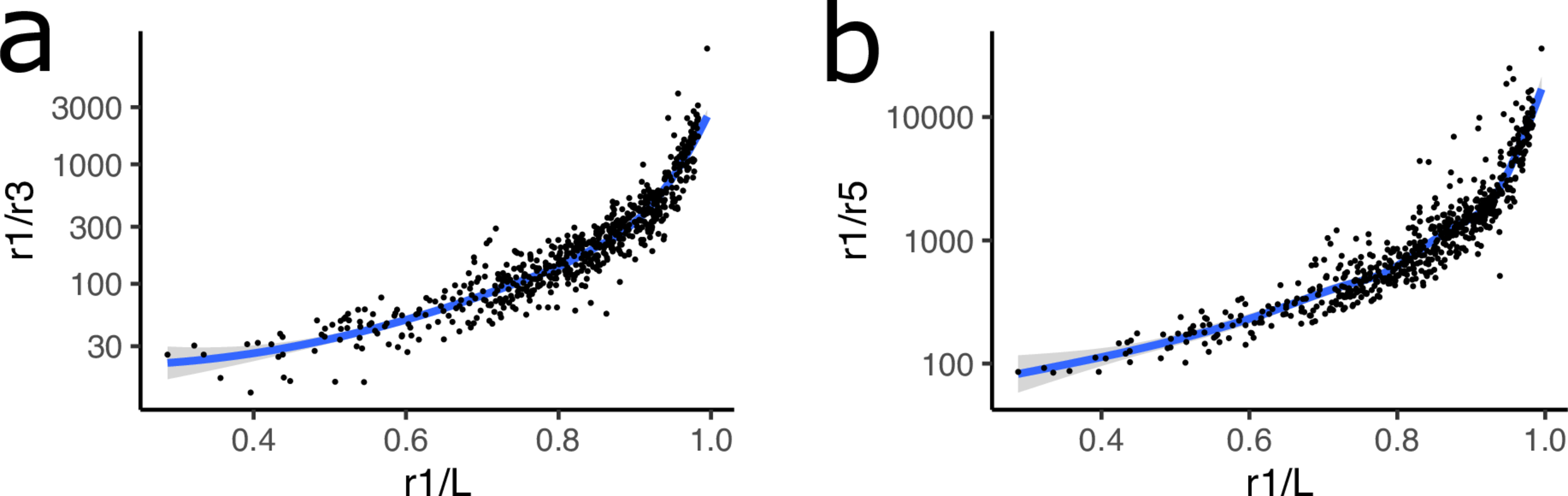

Supplement: S3 Fig — A: r1/r3 versus r1/L, B: r1/r5 versus r1/L. (TIF) [file pcbi.1009449.s004.tif]

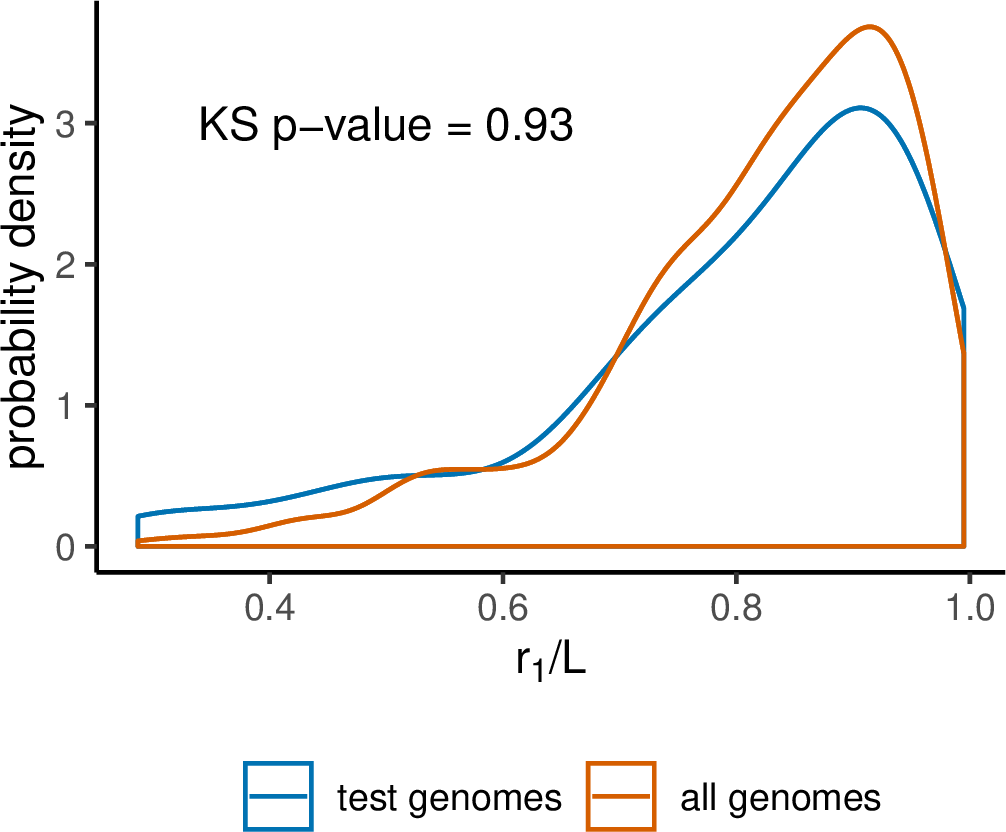

Supplement: S4 Fig — The p-value for the hypothesis that the distributions are different using two-sided Kolmogorov–Smirnov test is 0.93. Highly-repetitive genomes are slightly over-represented in the test set. (TIF) [file pcbi.1009449.s005.tif]

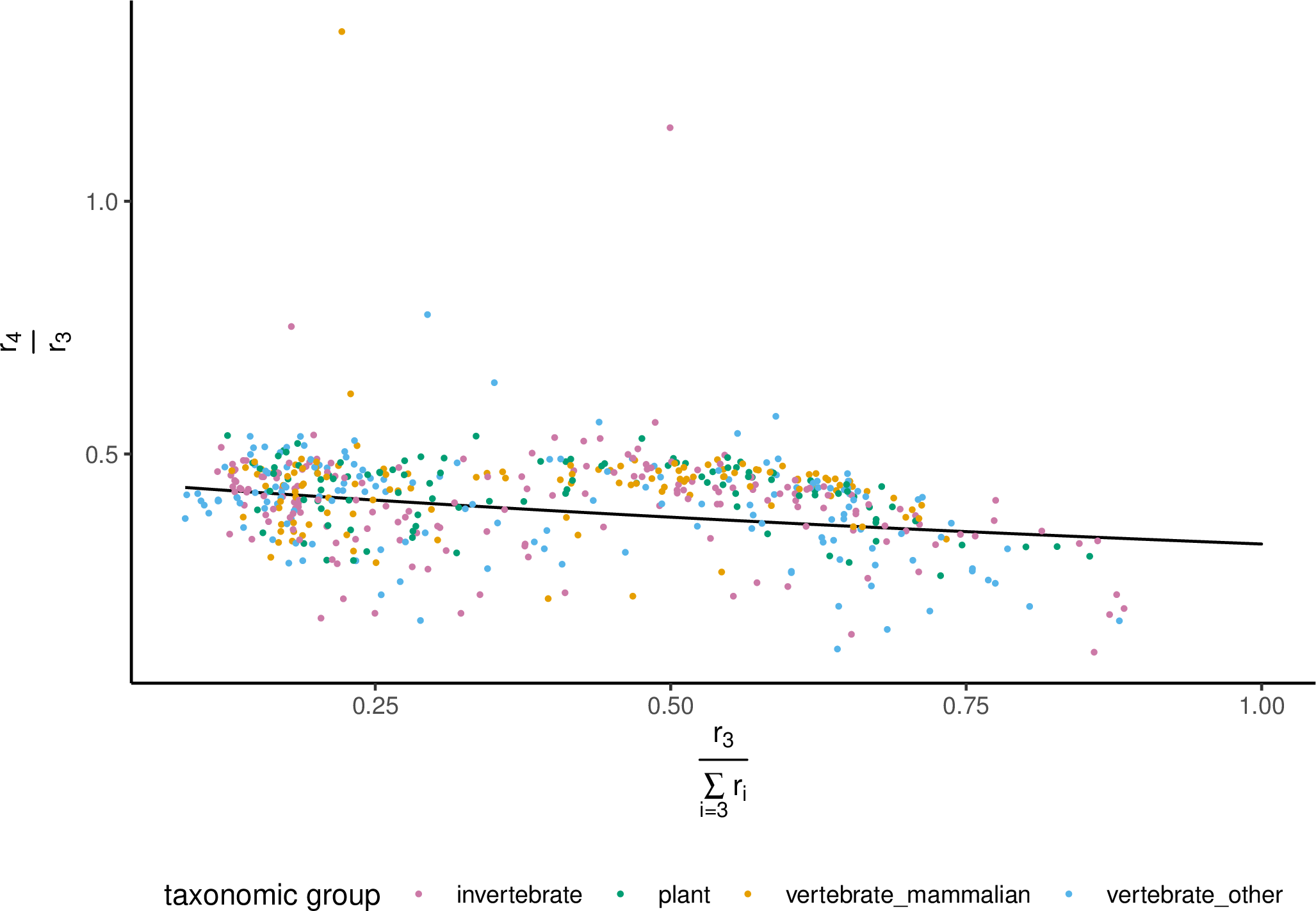

Supplement: S5 Fig — (TIF) [file pcbi.1009449.s006.tif]

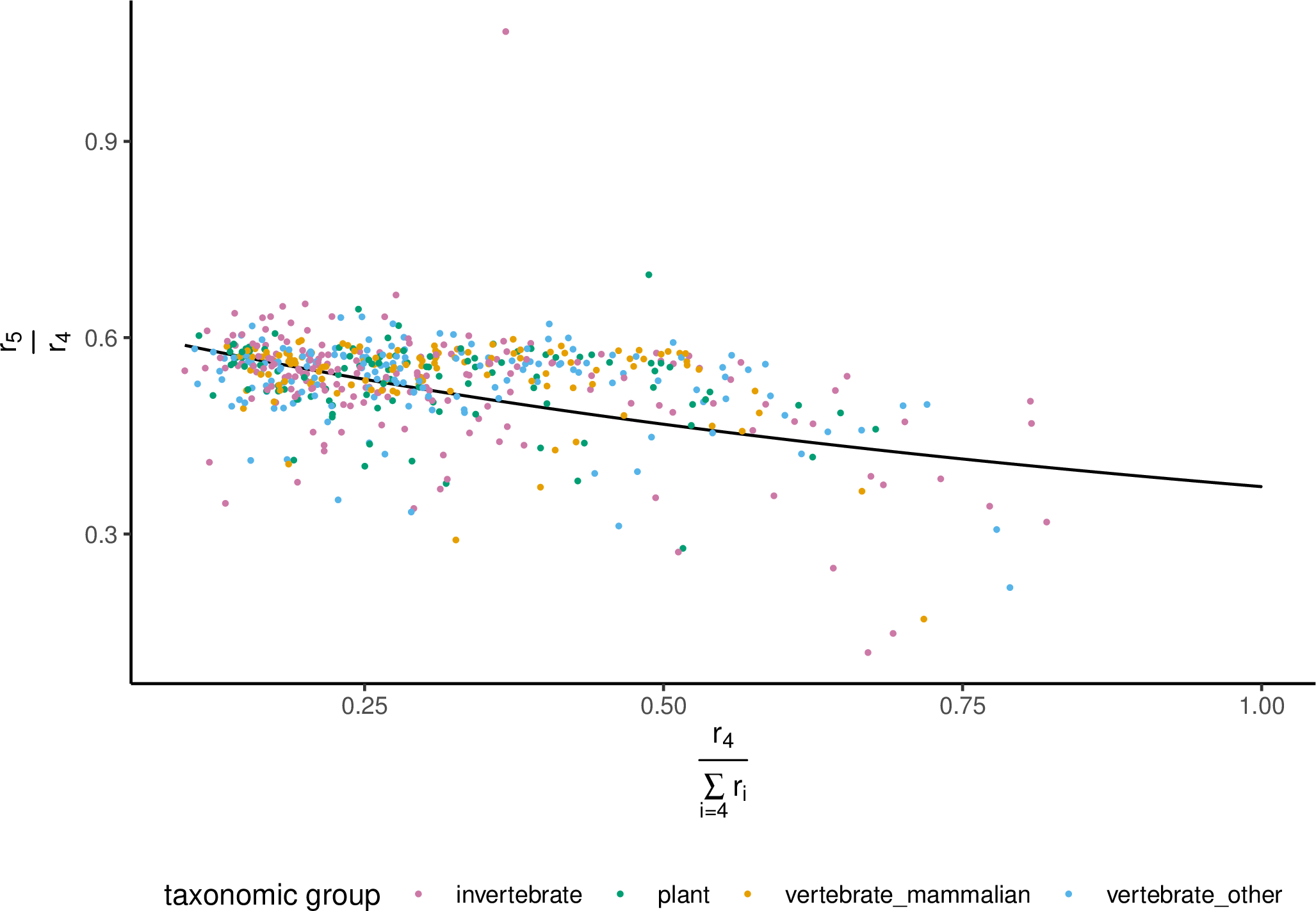

Supplement: S6 Fig — (TIF) [file pcbi.1009449.s007.tif]

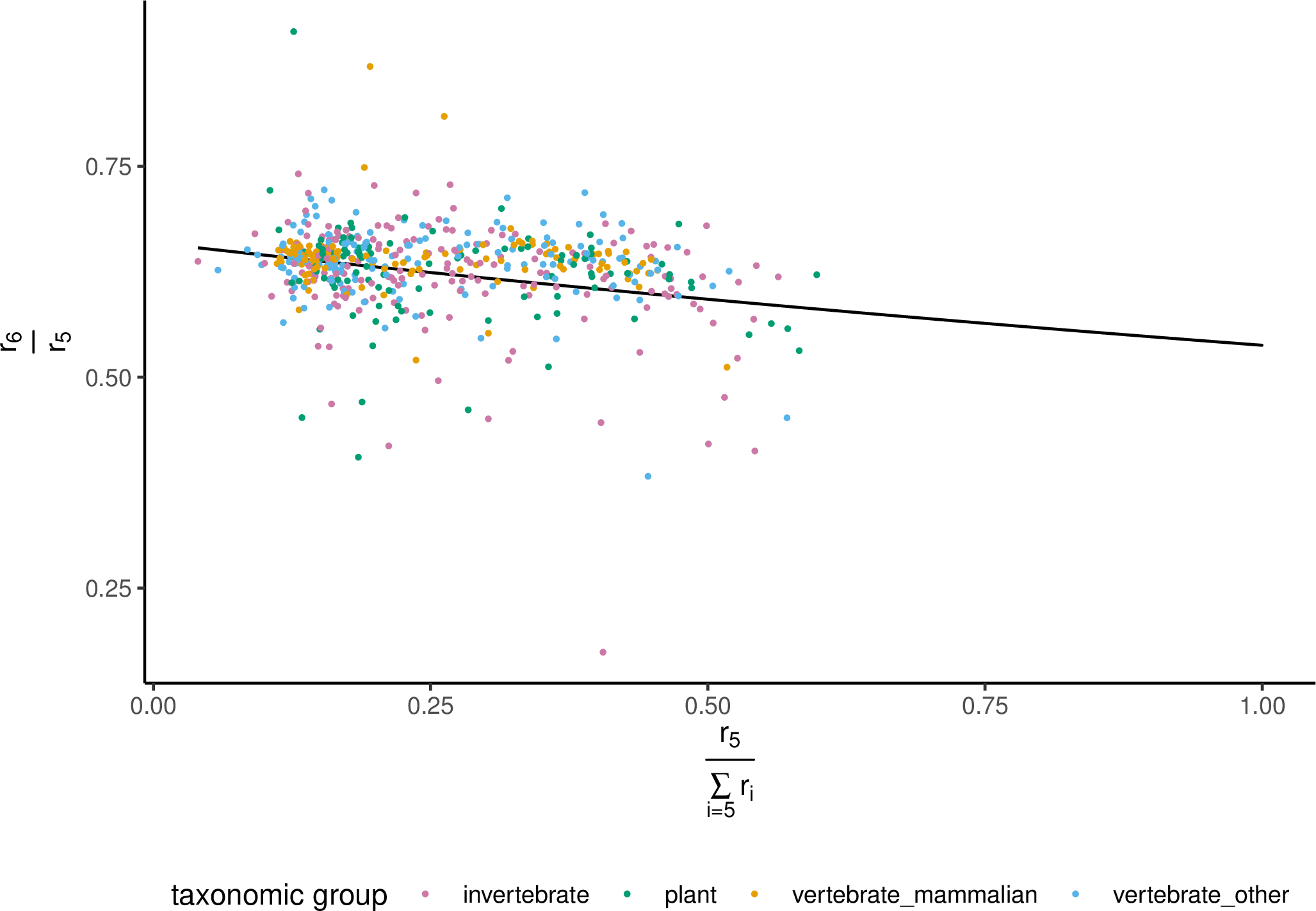

Supplement: S7 Fig — (TIF) [file pcbi.1009449.s008.tif]

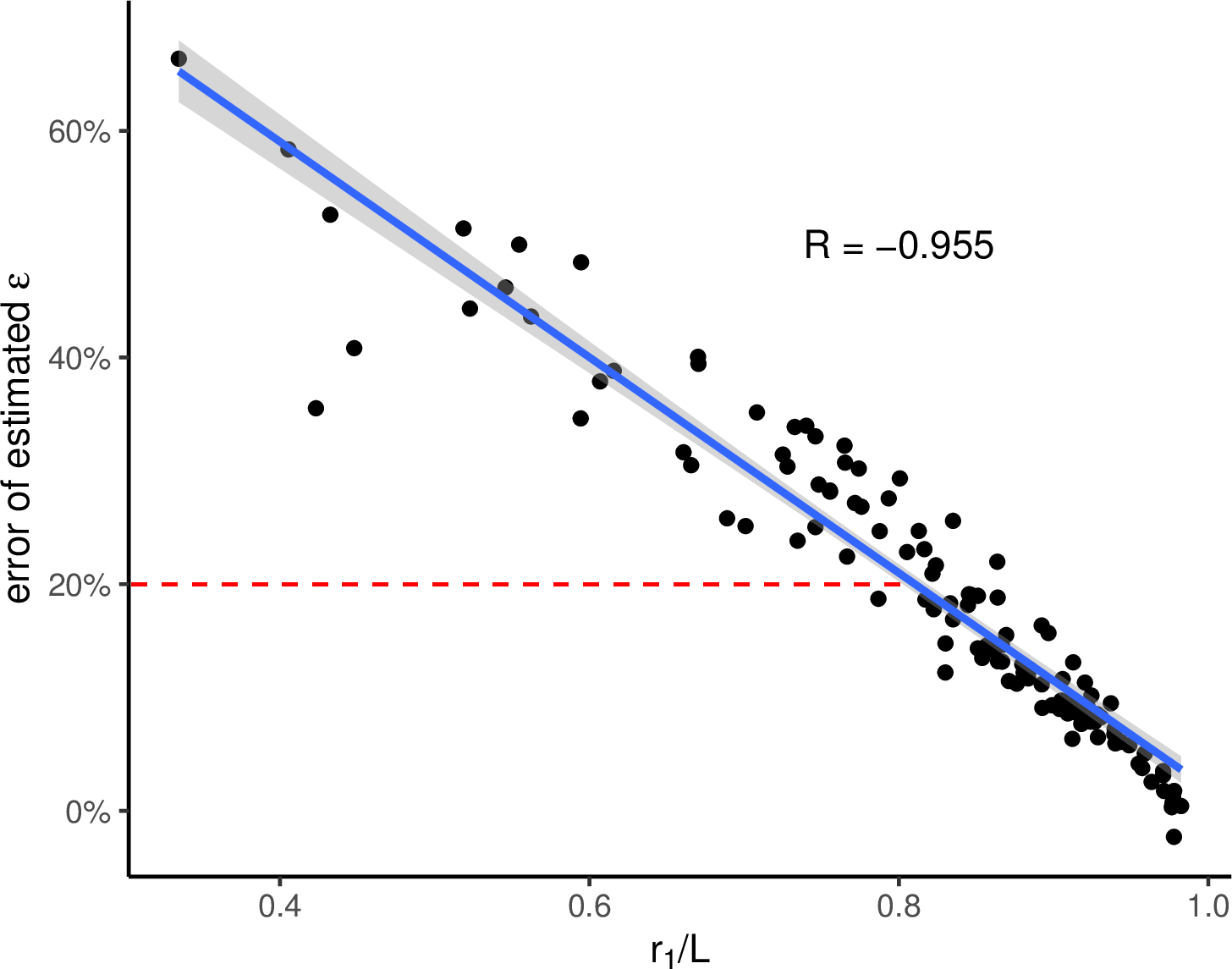

Supplement: S8 Fig — A subset of 120 training genomes were selected as the cross-validation set, and genome-skims were simulated at 1X coverage with 1% sequencing error rate. There is a strong correlation (R = −0.995) between the error in estimating ϵ and r1/L ratio. We capped the correction at 20% (red dashed line). (TIF) [file pcbi.1009449.s009.tif]

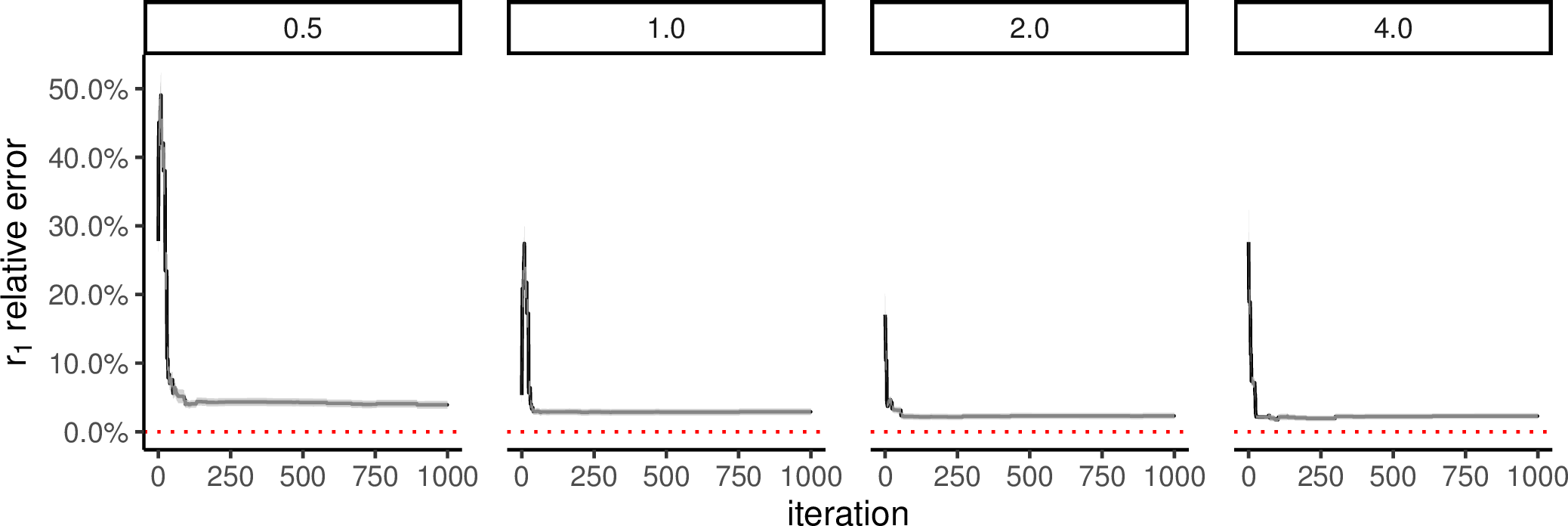

Supplement: S9 Fig — (TIF) [file pcbi.1009449.s010.tif]

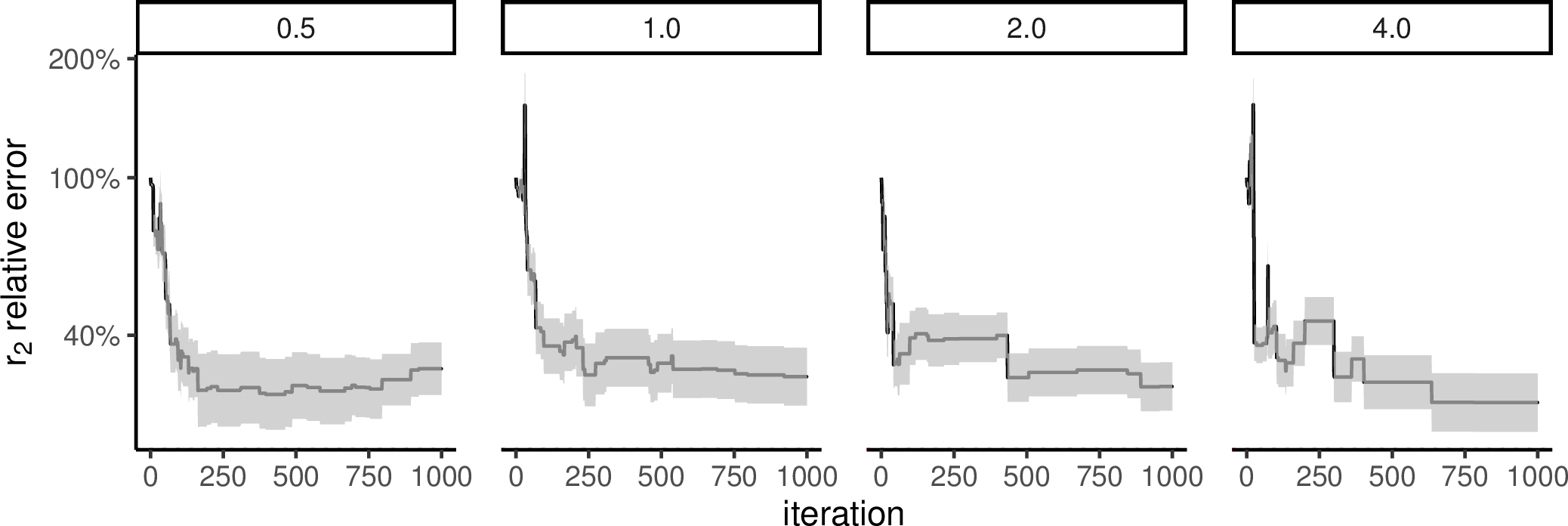

Supplement: S10 Fig — (TIF) [file pcbi.1009449.s011.tif]

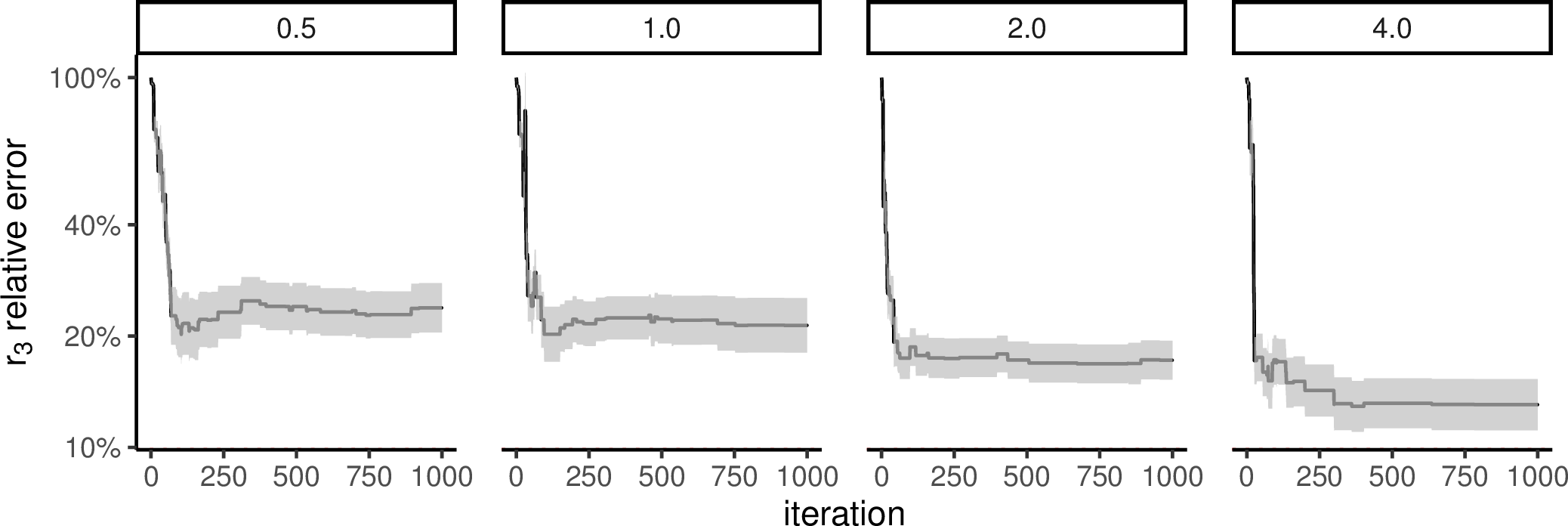

Supplement: S11 Fig — (TIF) [file pcbi.1009449.s012.tif]

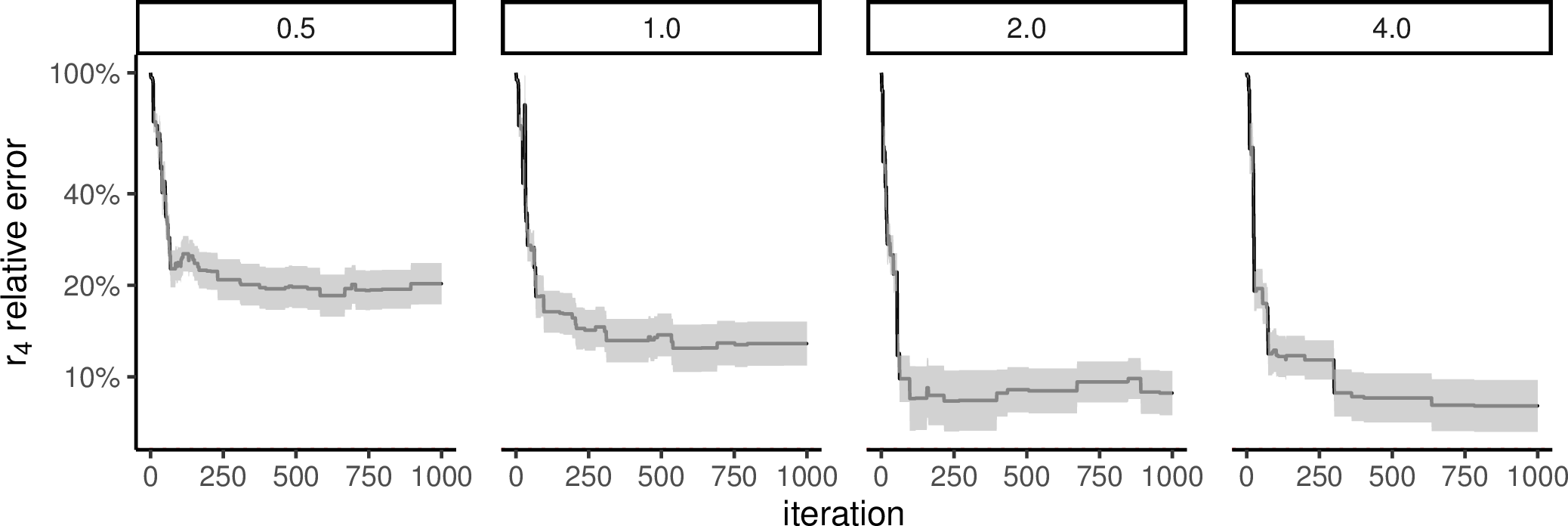

Supplement: S12 Fig — (TIF) [file pcbi.1009449.s013.tif]

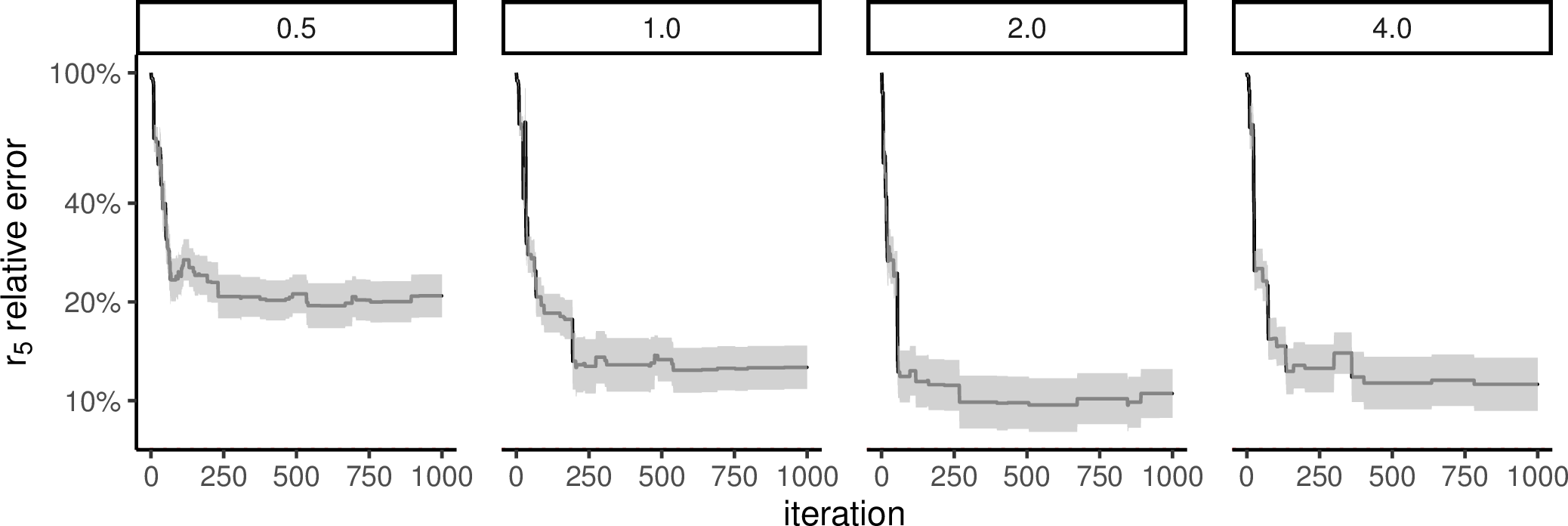

Supplement: S13 Fig — (TIF) [file pcbi.1009449.s014.tif]

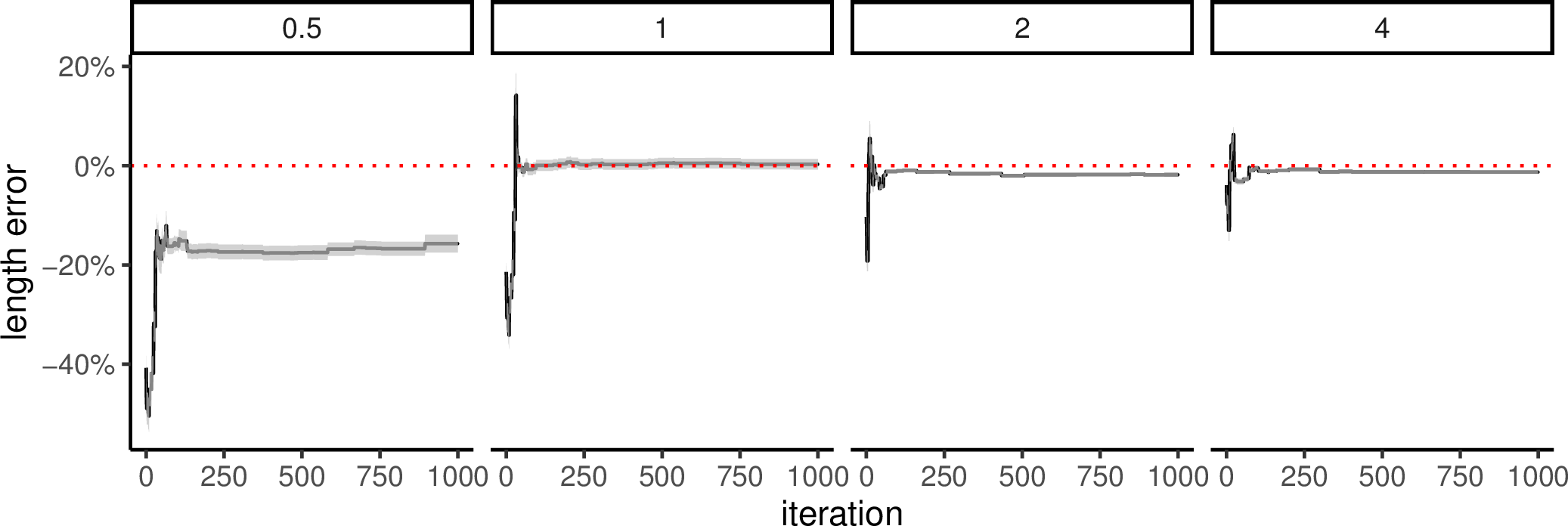

Supplement: S14 Fig — (TIF) [file pcbi.1009449.s015.tif]

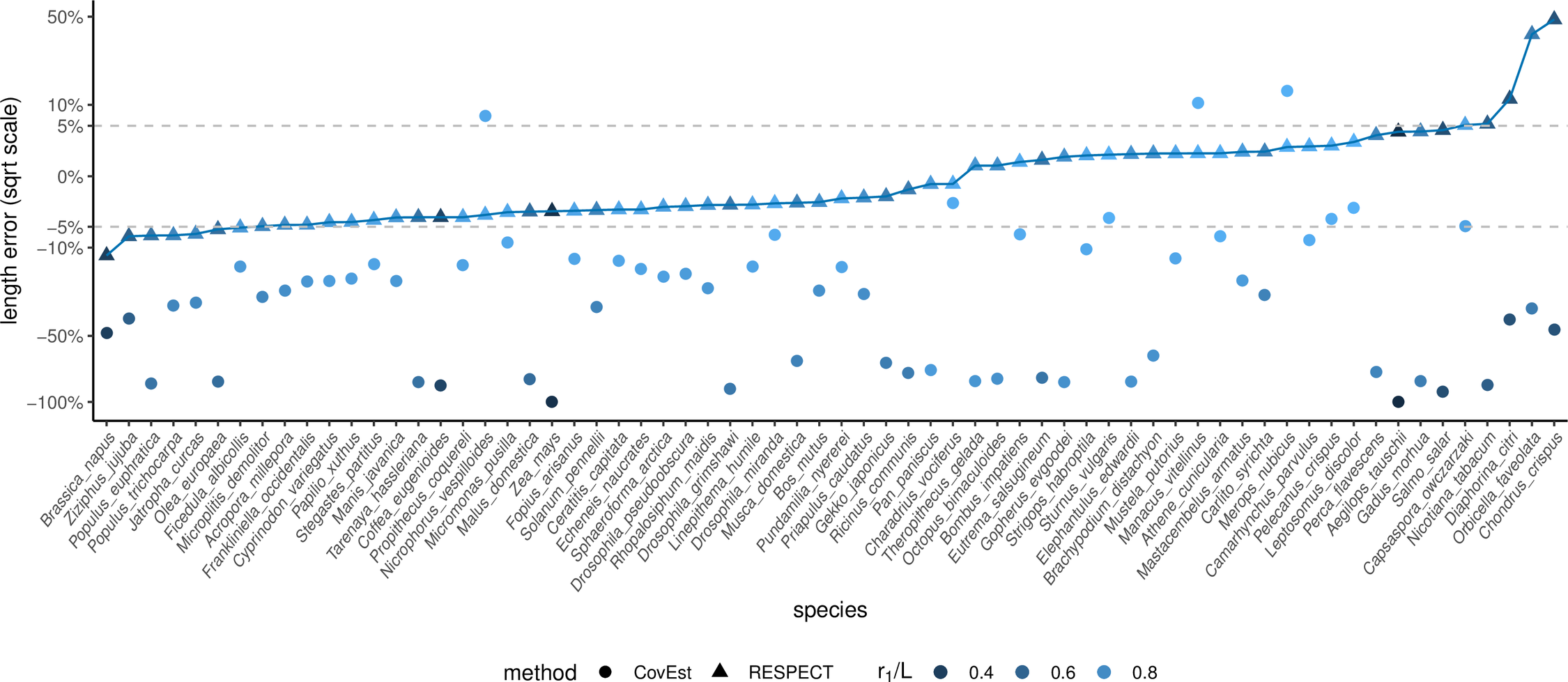

Supplement: S15 Fig — The coverage is 1X, and the y-axis is in square-root scale. The sign of error indicates overestimation or underestimation. The dashed lines mark the region that the absolute value of error is less than 5%. (TIF) [file pcbi.1009449.s016.tif]

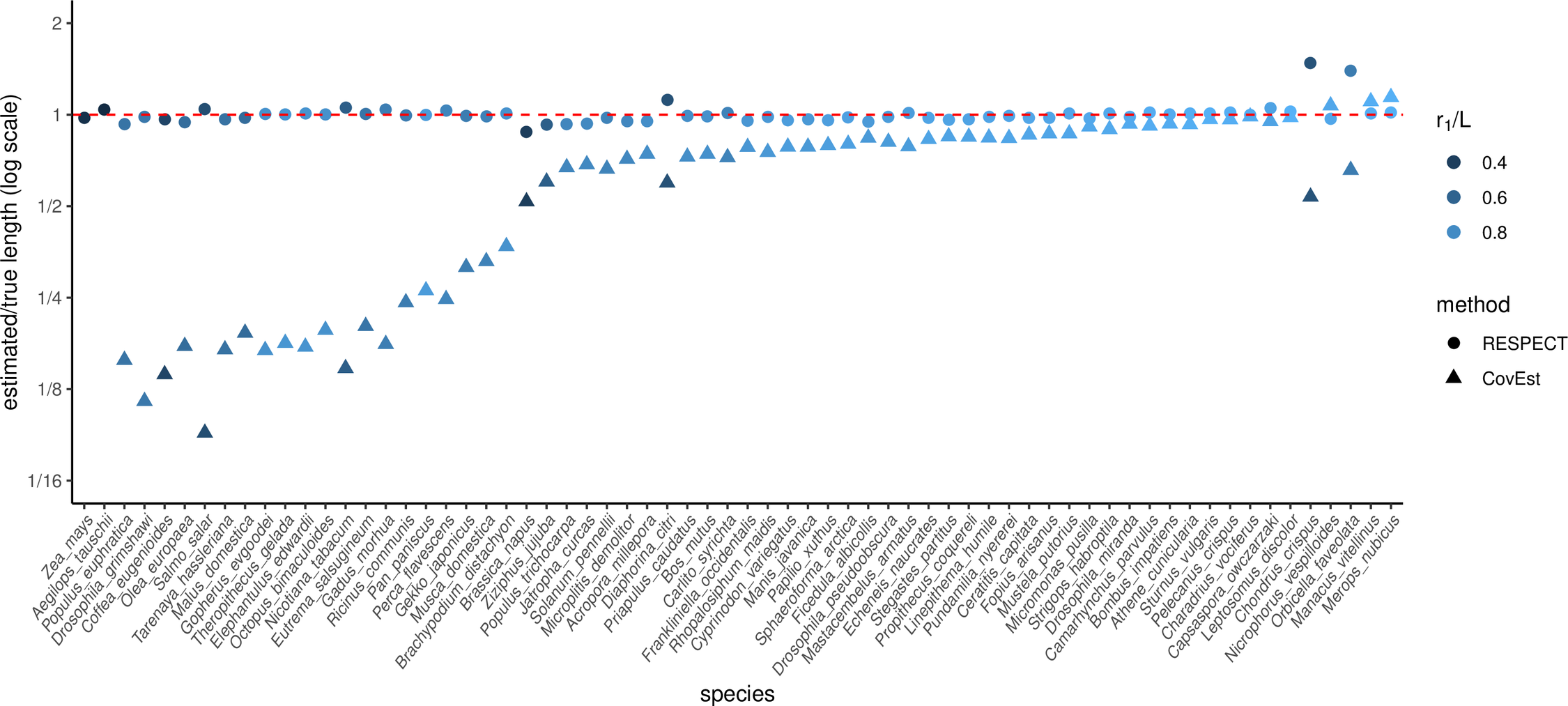

Supplement: S16 Fig — Comparing RESPECT and CovEst over 66 test species with genomes skimmed at 1X coverage. The y-axis is plotted in log scale, and the red dashed line at y = 1 is the grand truth (no error). Two genomes (A. tauschii (0.002) and Z. mays (0.003)) that CovEst had extremely low estimated to true ratios were removed to improve readability. (TIF) [file pcbi.1009449.s017.tif]

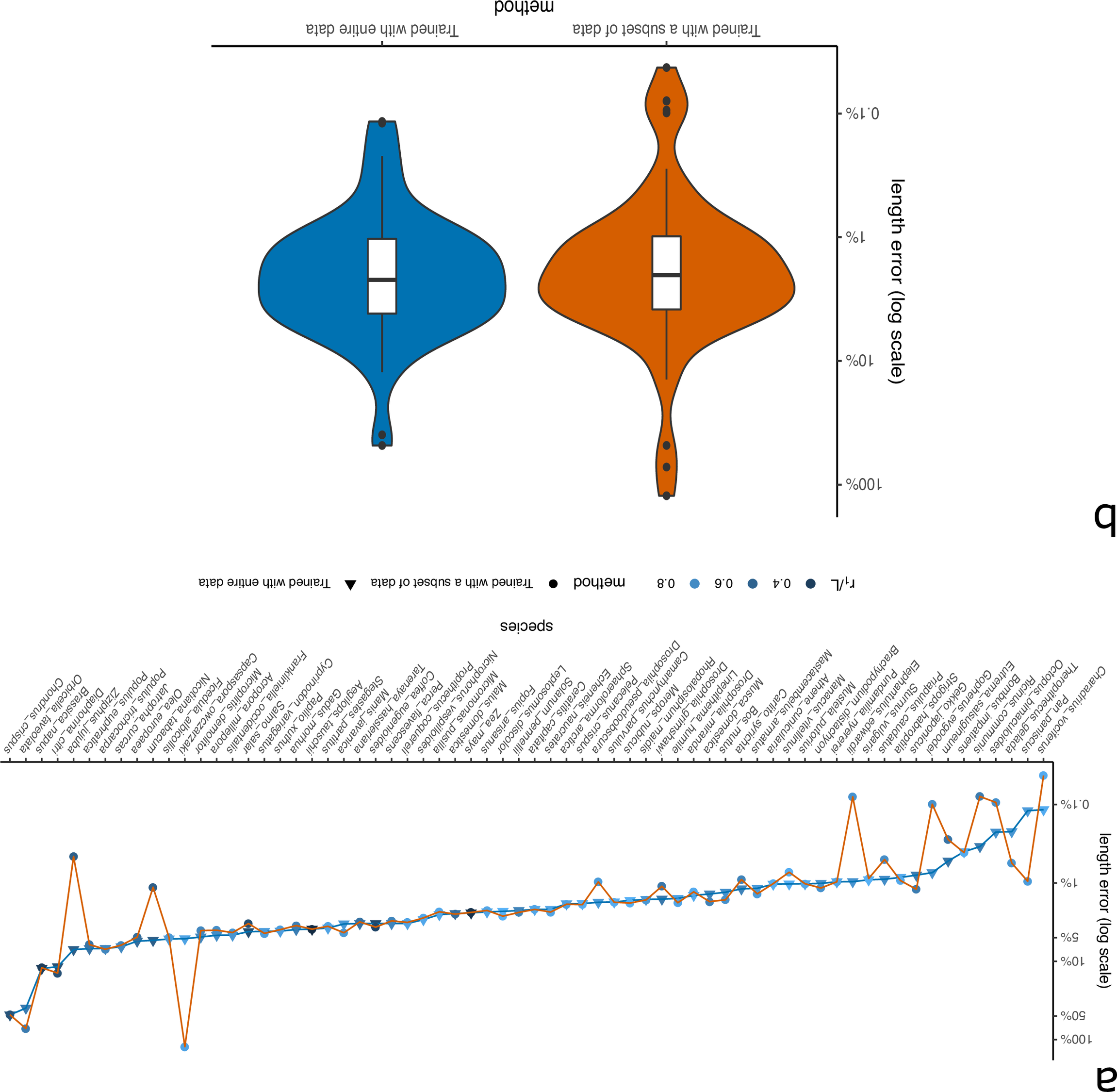

Supplement: S17 Fig — RESPECT was trained on a subset of genomes (50 of 129 mammalian genomes and 50 of 195 invertebrate genomes were removed), and the error plotted (circles) along with the error on the original training set (triangles). A: The error per genome is plotted in log scale on the y-axis. B: The distribution of error values with RESPECT trained on the subset (blue) and the entire data set (red). (TIF) [file pcbi.1009449.s018.tif]

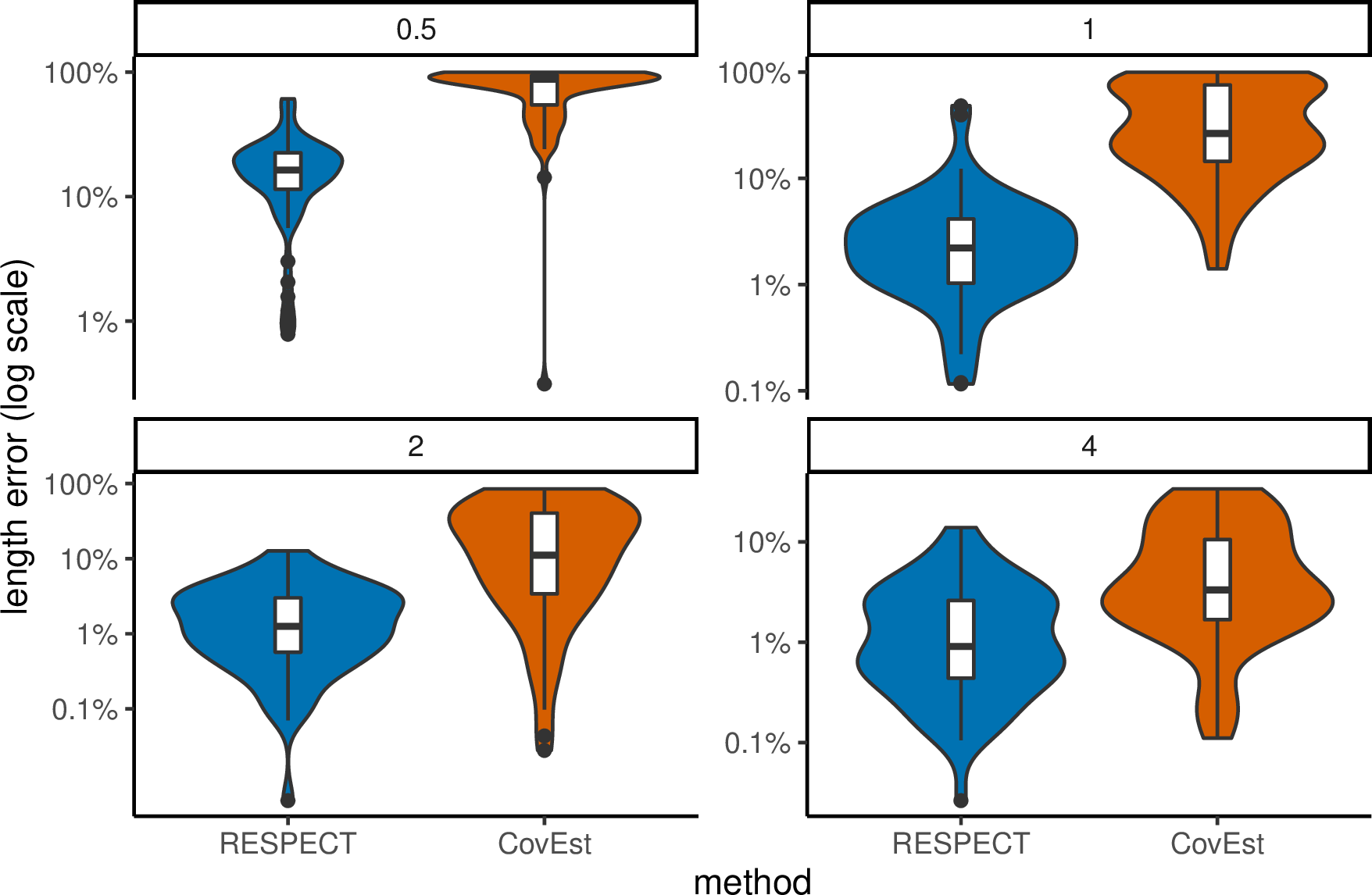

Supplement: S18 Fig — The distribution of error made by RESPECT and CovEst in estimating the length of 66 test genomes skimmed at 0.5X, 1X, 2X, and 4X coverage. The y-axis is plotted in log scale. (TIF) [file pcbi.1009449.s019.tif]

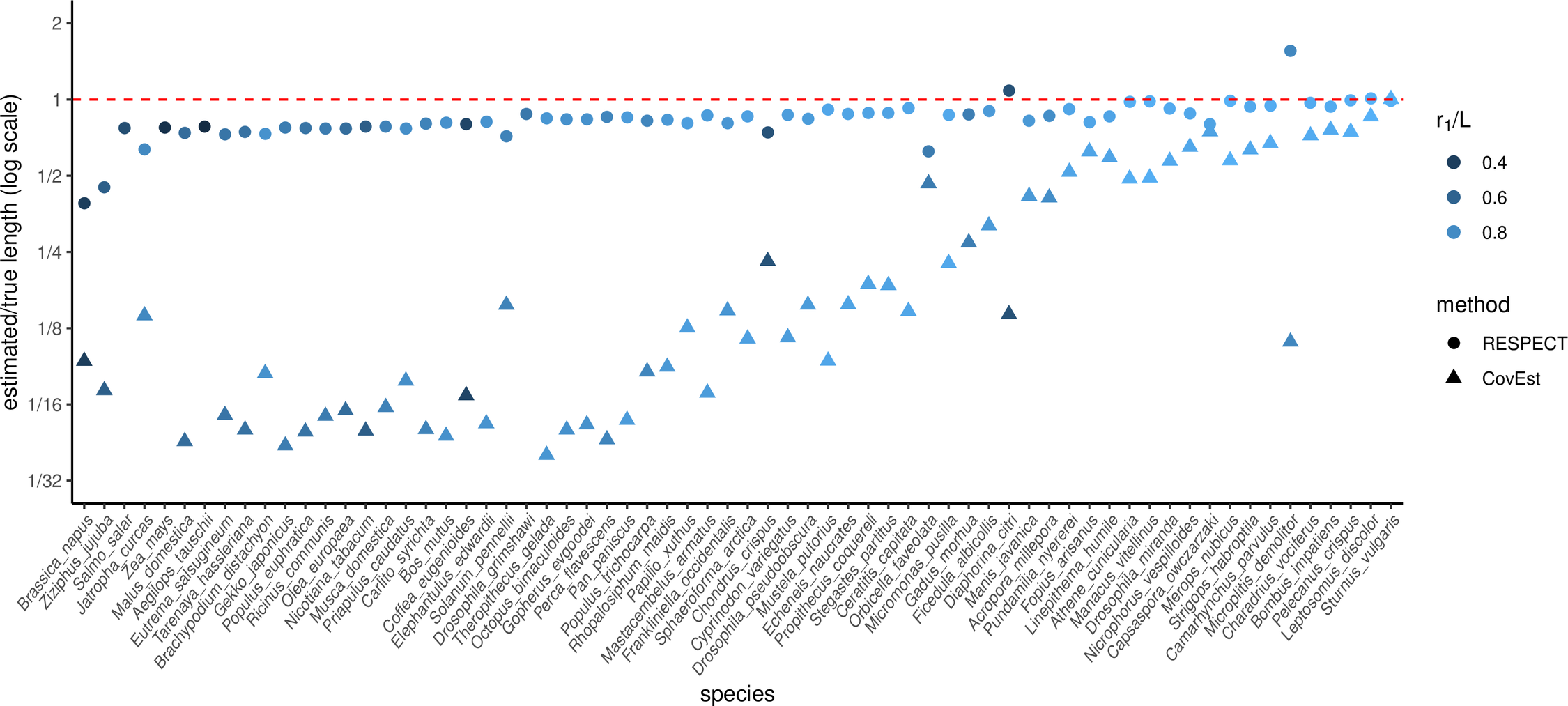

Supplement: S19 Fig — Comparing RESPECT and CovEst over 66 test species with genomes skimmed at 0.5X coverage. The y-axis is plotted in log scale, and the red dashed line at y = 1 is the grand truth (no error). Four genomes (D. grimshawi (0.0004), S. salar (0.0006), A. tauschii (0.0012), and Z. mays (0.0016)) that CovEst had extremely low estimated to true ratios were removed to improve readability. (TIF) [file pcbi.1009449.s020.tif]

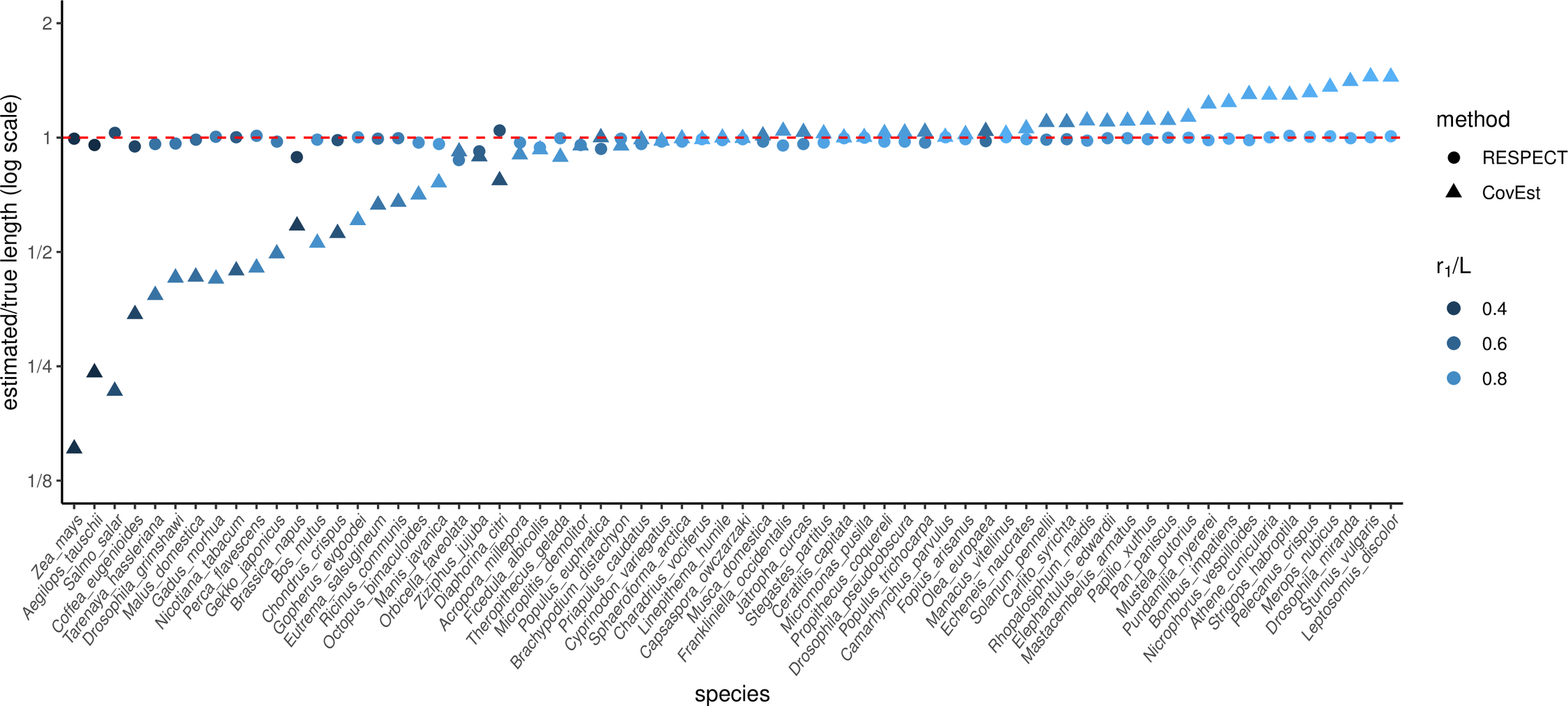

Supplement: S20 Fig — Comparing RESPECT and CovEst over 66 test species with genomes skimmed at 2X coverage. The y-axis is plotted in log scale, and the red dashed line at y = 1 is the grand truth (no error). (TIF) [file pcbi.1009449.s021.tif]

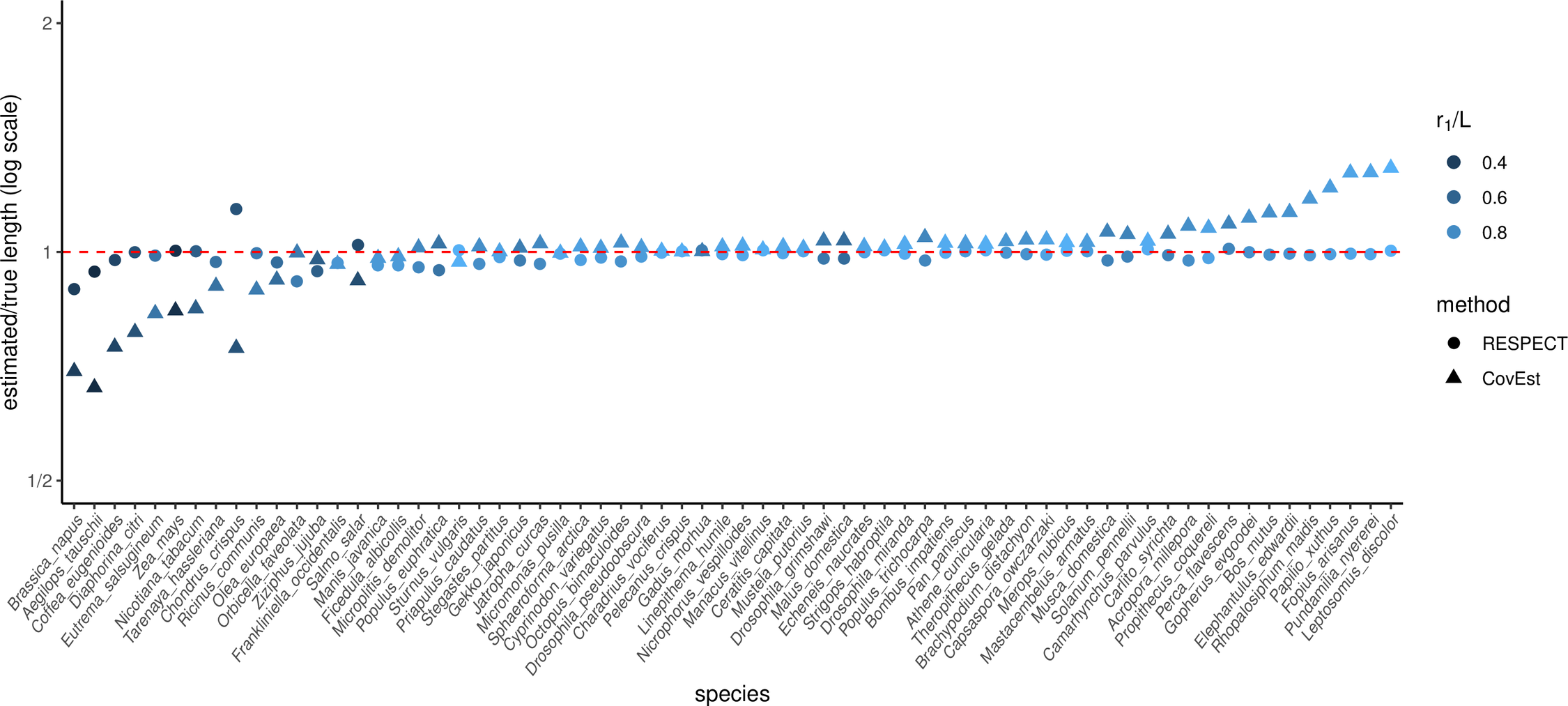

Supplement: S21 Fig — Comparing RESPECT and CovEst over 66 test species with genomes skimmed at 4X coverage. The y-axis is plotted in log scale, and the red dashed line at y = 1 is the grand truth (no error). Four genomes (D. grimshawi (0.0004), S. salar (0.0006), A. tauschii (0.0012), and Z. mays (0.0016)) that CovEst had extremely low estimated to true ratios were removed to improve readability. (TIF) [file pcbi.1009449.s022.tif]

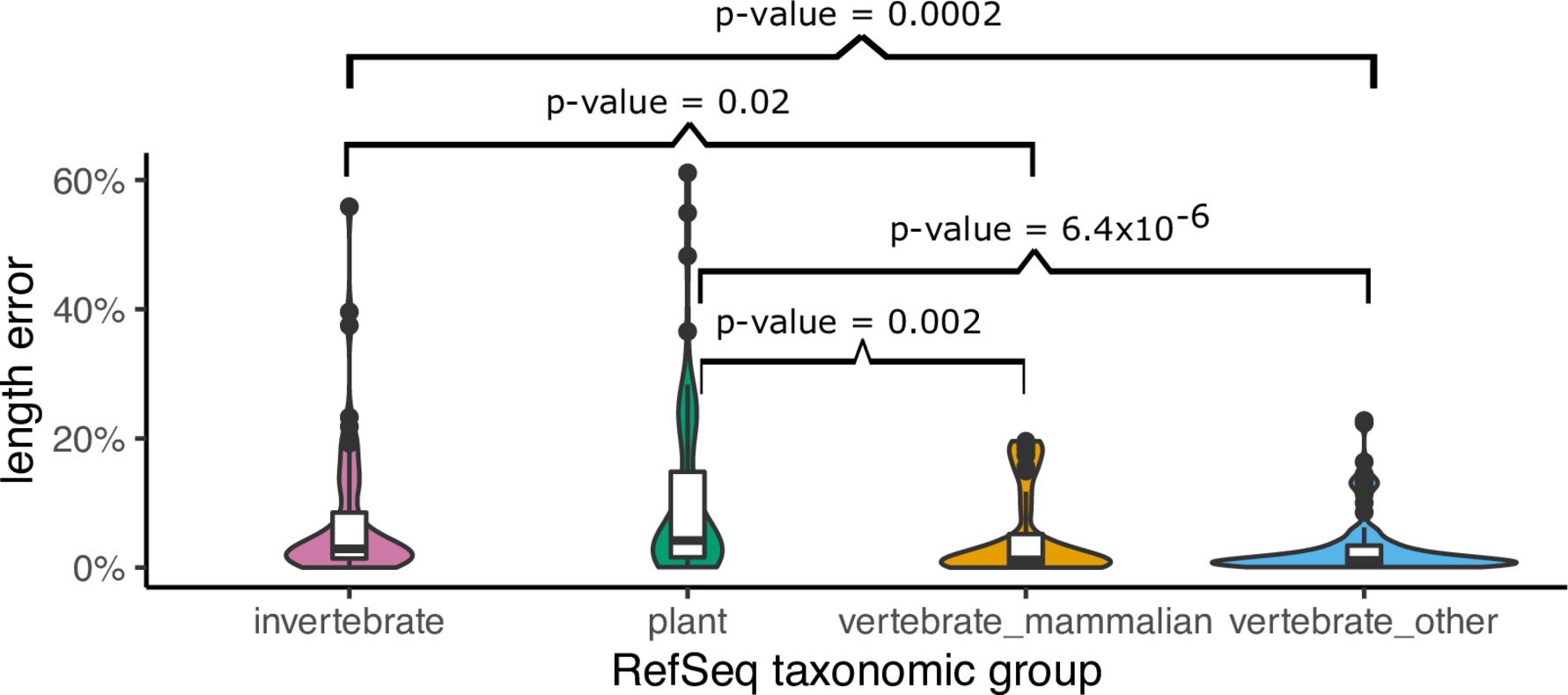

Supplement: S22 Fig — Significant p-values (0.05 threshold) computed using Mann-Whitney U test are added to the plot. Plants and invertebrates have higher error rates compared to vertebrates species in our test dataset. (TIF) [file pcbi.1009449.s023.tif]

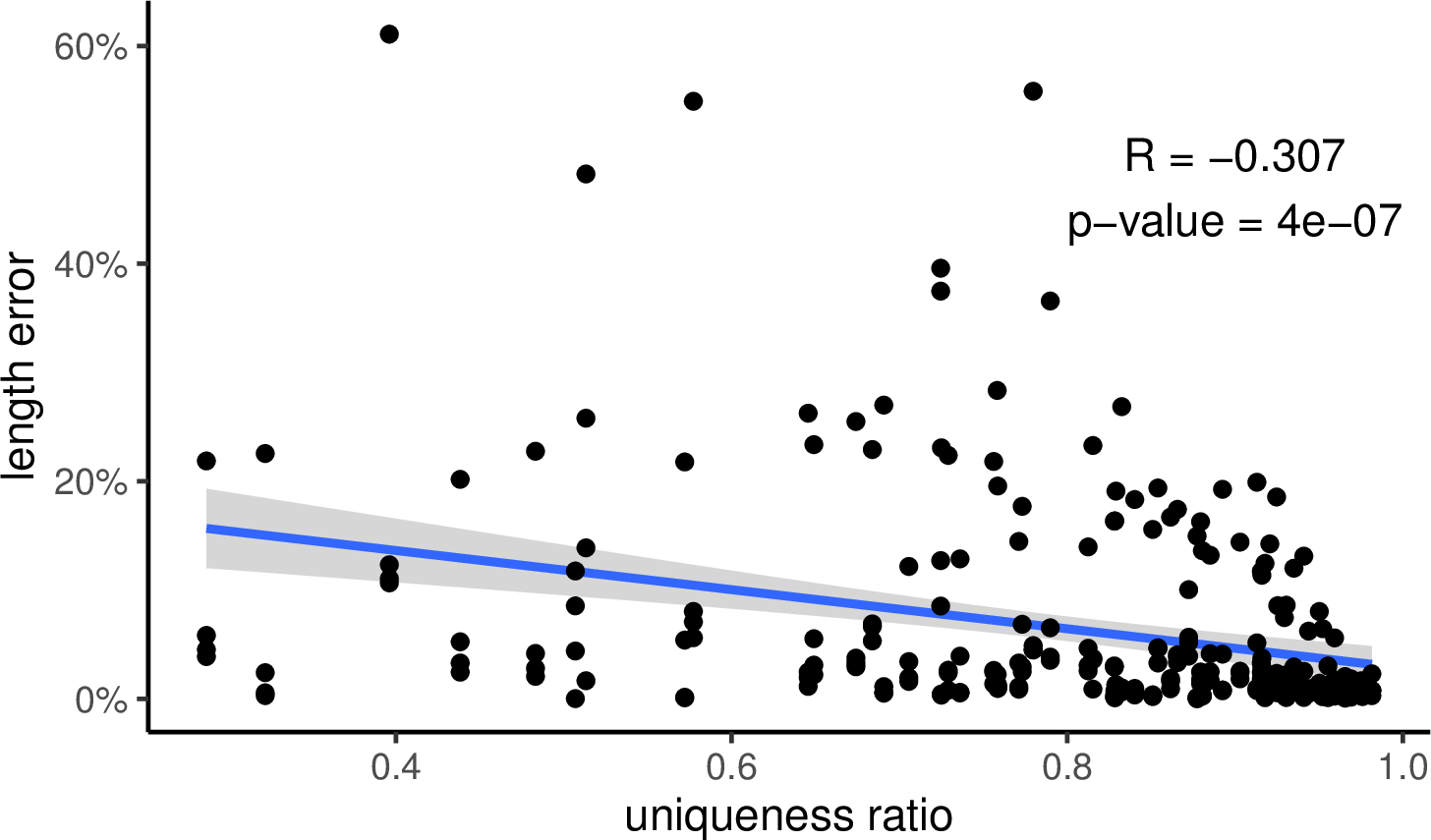

Supplement: S23 Fig — Negative correlation between RESPECT’s error and uniqueness ratio of the genome. (TIF) [file pcbi.1009449.s024.tif]

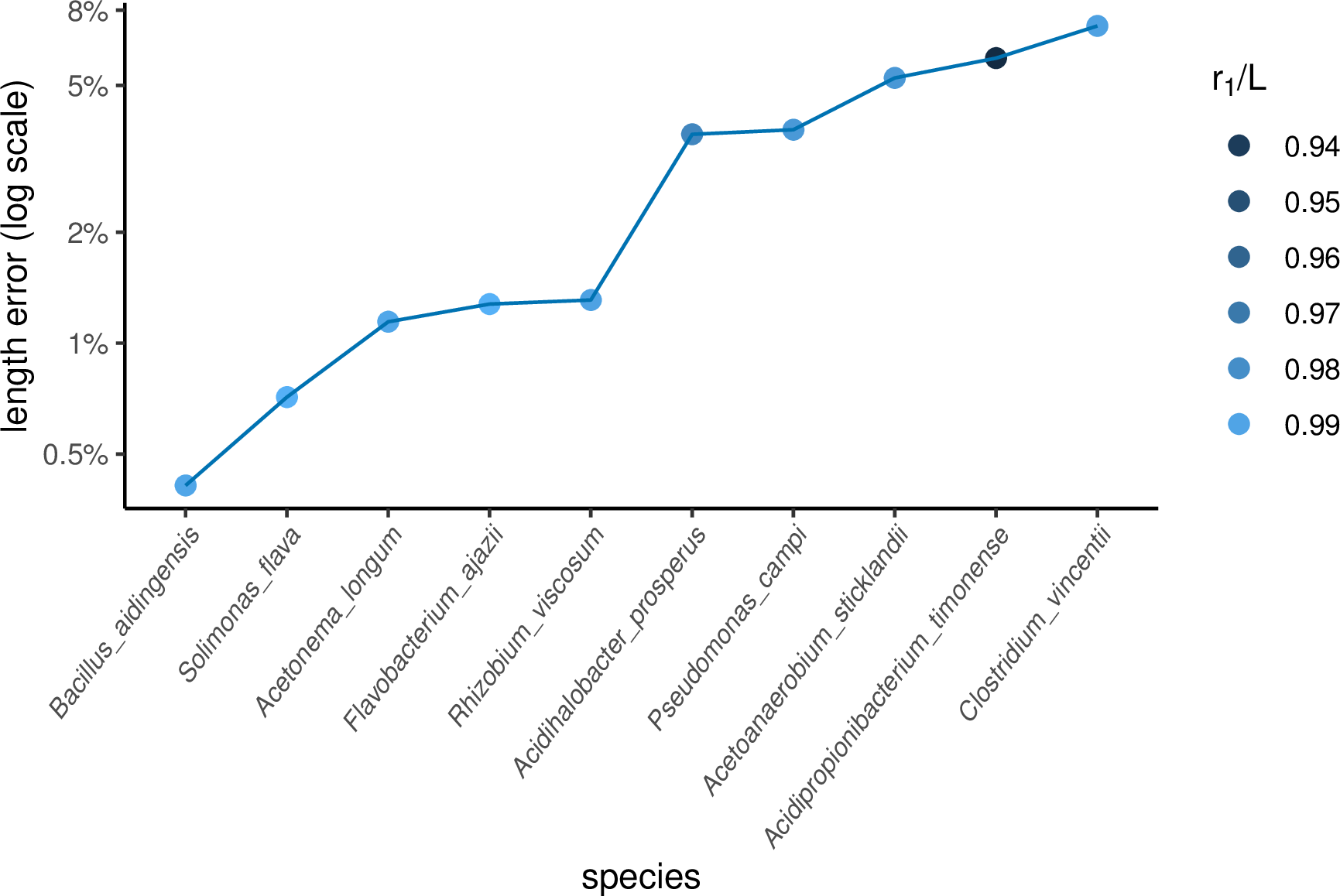

Supplement: S24 Fig — The 10 bacterial genomes were selected at random from RefSeq and genome-skims were simulated at 1X coverage. The relative error of the estimated length is plotted in log scale on the y-axis. (TIF) [file pcbi.1009449.s025.tif]

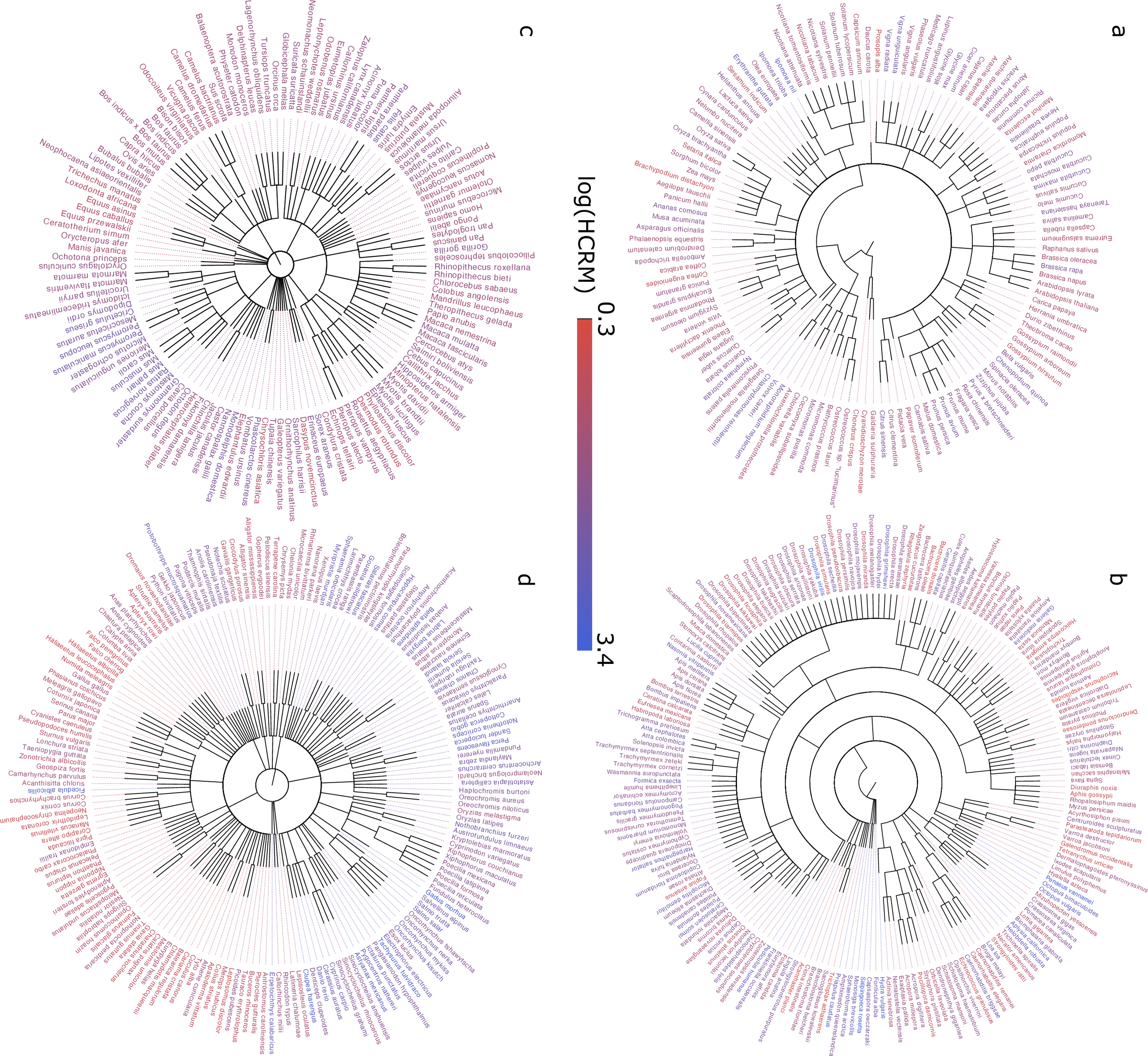

Supplement: S25 Fig — Colors are based on logarithm of HCRM values for each genome. A: Plants, B: Invertebrates, C: Mammals, D: Other vertebrates. (TIF) [file pcbi.1009449.s026.tif]

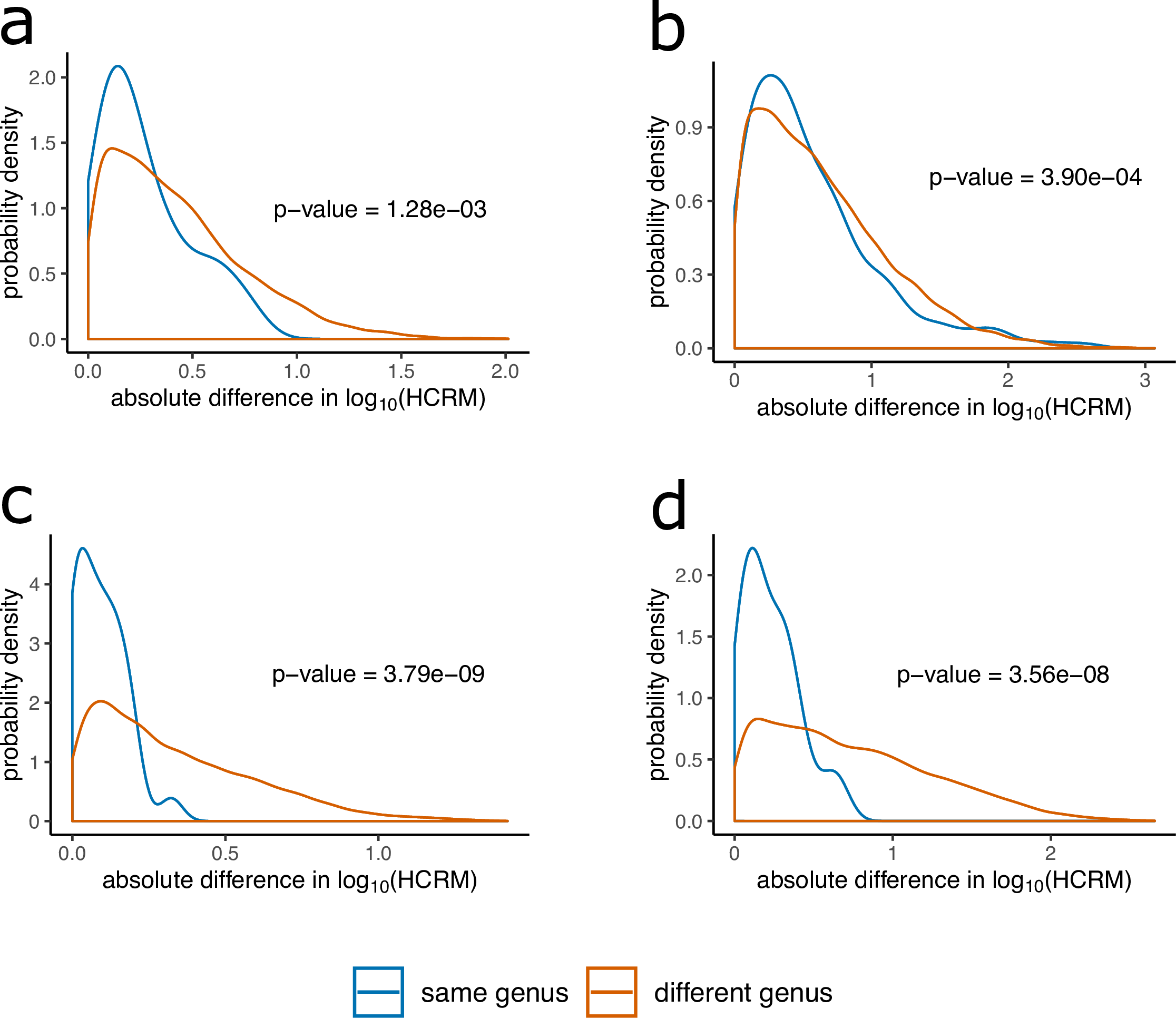

Supplement: S26 Fig — A: Plants, B: Invertebrates, C: Mammals, D: Other vertebrates. (TIF) [file pcbi.1009449.s027.tif]

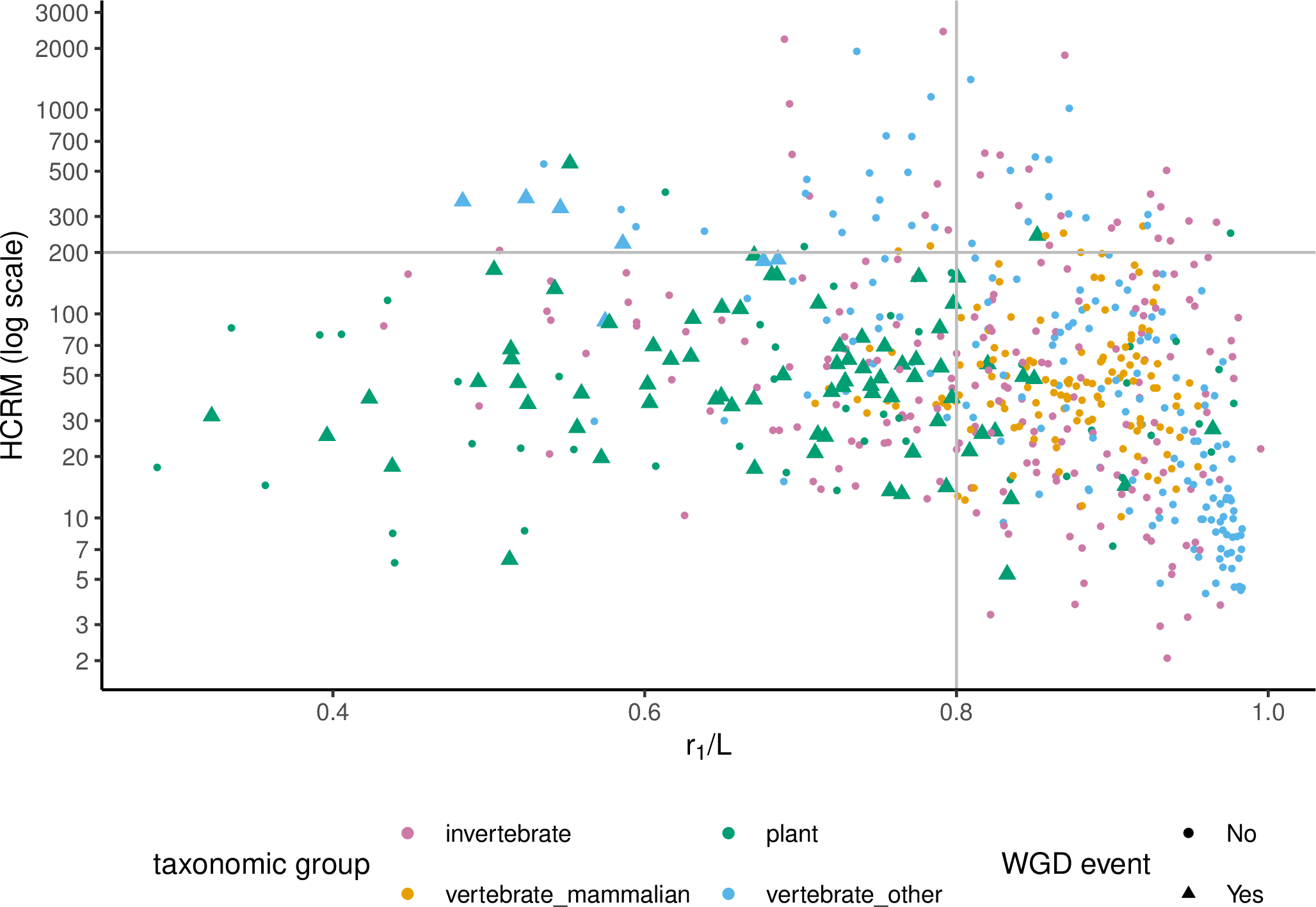

Supplement: S27 Fig — HRCM values are computed directly from the genome assemblies. (TIF) [file pcbi.1009449.s028.tif]

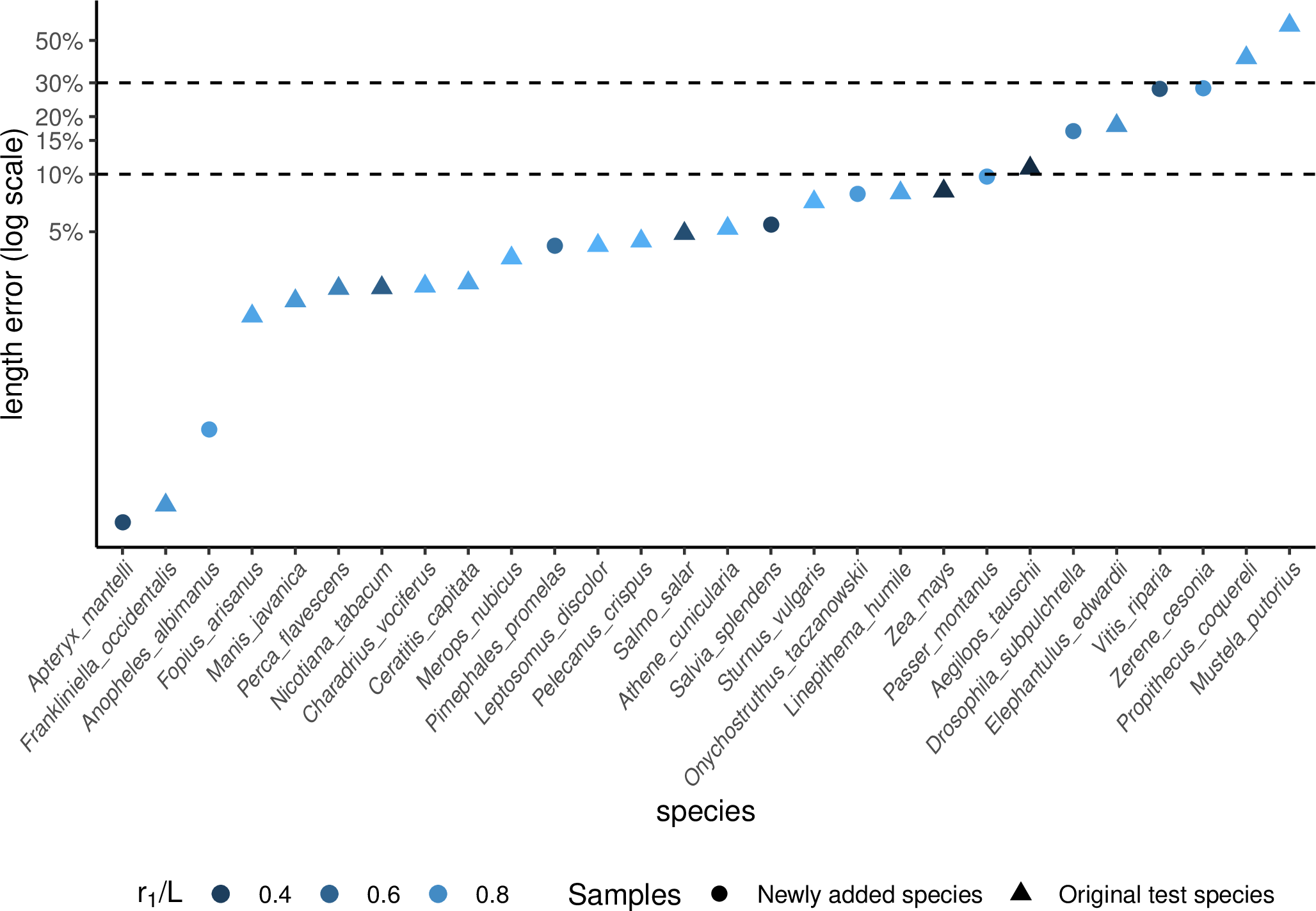

Supplement: S28 Fig — RESPECT was test on 10 new samples (chosen at random) made available since the original submission of the manuscript. One of the samples was removed during the preprocessing due to high duplication rate. The results for the remaining 9 samples are plotted along with the original test species. Two newly added samples with high error are Z. cesonia and V. riparia. RESPECT overestimates their genome length by %28. It could be the case that the assemblies are missing some repetitive sequences (especially V. riparia which a has highly repetitive genome), considering that for both species there is a gap between reported total sequence length and total ungapped length. (TIF) [file pcbi.1009449.s029.tif]
