# Supplementary material for: Estimating repeat spectra and genome length from low-coverage genome skims with RESPECT
Source: PLoS Comput Biol. 2021 Nov 15;17(11):e1009449. doi: 10.1371/journal.pcbi.1009449 (PMC8629397; doi:10.1371/journal.pcbi.1009449)
Supplement: Attachment — Submitted filename: RESPECT-reviews-response.docx [file pcbi.1009449.s030.docx]

**Reviewer #1**:

The authors present a new tool called RESPECT to perform estimation of genome length, coverage and repeat content from high-throughput shotgun sequencing reads. They show that their tool provides improved performance compared to other similar alternatives (namely CovEst). Although this is a tool with a highly specific utility, the authors have done a good job in describing the rationale behind their approach and providing sufficient details on how their method works. I think this will be a valuable contribution to the field. I have a few comments that I believe would make the tool even more useful and convincing.

1) In the manuscript, RESPECT is only evaluated against eukaryotic genomes. Given that the majority of sequence repositories are dominated by prokaryotic sequences, it would be very useful to understand how their tool behaves with bacterial genomes as well, at least in predicting genome length and coverage. In principle, I see no obvious reason why it should not work, aside from the fact that bacterial genomes are much less repetitive. This would also provide a more diverse sequence set to understand if the accuracy of their method is biased to particular taxonomic groups.

**Response.** To address the reviewer's point, we have tested RESPECT on 10 bacterial species (randomly selected from RefSeq). Genome skims at 1X coverage were simulated for each species and then RESPECT was run to estimate the genome length. The relative error in estimating the genome length is computed based on the length of available assemblies (Figure S24). As pointed out by the reviewer, the bacterial genomes are fairly simple in terms of high uniqueness ratio and so RESPECT is able to make accurate estimations. As RESPECT is developed to specifically work at low coverages under genome skimming scenarios, and sequencing depth is usually not a limiting factor for small bacterial genomes, we have added the results as a separate supplemental figure to be consistent with the overall narrative of the manuscript.

**Figure S24**

2) For Fig. 3 it would be useful to evaluate the length error by taxonomy. There seems to be a big range in the accuracy of their method (from 0.1% to 50%). Is their algorithm better at predicting the length of certain taxa as opposed to others?

**Response.** We have compared the distribution of length estimation error across 4 major taxonomic groups within our test dataset (Figure S22). The error is higher among plants and invertebrates when their distribution of error is compared to vertebrates (Mann-Whitney U test, p-value<0.05). However, there is no statistical difference between mammals and other vertebrates. Relatively higher error rates in some plant and invertebrate species is consistent with higher prevalence of repetitive sequences among these species (Figure 1b). In fact there is a statistically significant (negative) correlation between length estimation error and uniqueness ratios among our test data (Figure S23).

**Figure S22**

**Figure S23**

3) I wonder if it would be possible to further improve their genome length predictions by taking into account prior knowledge on the corresponding taxa. For instance, the authors could perform a first-pass k-mer assignment of the sequence data to a database like RefSeq to determine which taxonomic group is dominated in the data. Based on this match, they would deduce that the predicted genome length would likely sit between a certain range determined by the known genomes (e.g., in RefSeq) within that taxa. This could potentially resolve some cases where the error rates are >5-10%.

**Response.** In this paper we have addressed the problem of estimating genomic parameters and assume that the taxon is known. In fact, we have a method ([Skmer](https://genomebiology.biomedcentral.com/articles/10.1186/s13059-019-1632-4)) that can be used for sample identification, and also a method ([APPLES](https://academic.oup.com/sysbio/article/69/3/566/5572672)) for phylogenetic placement, which can be used if the sample identity is unknown. However, we do not see an easy way to predict genome length using taxonomic information as there is a large variation in genome length within each taxonomic group.

On the other hand, the uniqueness ratio of the genome seems to be correlated with error (Figure S23), and there is some correlation in uniqueness ratio within genera (Figure S2), so future work can focus on this correlation and use the taxonomic information to compute confidence intervals on the estimated parameters. We thank the reviewer for this comment.

4) I have some concerns regarding the test/training datasets used to evaluate their tool. I understand the authors used 66 species as the test dataset, representing the diversity of RefSeq. However, how phylogenetically balanced were the remaining 556 genomes used for training? I imagine there were clear biases for certain taxonomic groups. How did the authors account for this?

**Response.** We took *every* eukaryotic genome available on RefSeq at the time of preparation (December 2019) and used them to train our model (excluding the test species), so at least in that sense, there was no bias. To address the reviewer’s concern about the phylogenetic balance of our training data, we removed 50 of 129 total mammalian genomes and also 50 of 195 invertebrate genomes and re-trained the model (Figure S17a). While the results are data dependent, the difference in the error is not statistically significant (Figure S17b). Also, adding more species reduces the maximum error in length estimation (Figure S17b). Therefore, as more assemblies become available, the trained model becomes more representative of the diversity of genomes in nature and worst case error is reduced.

**Figure S17a**

**Figure S17b**

5) The number of public, sequenced datasets used for benchmarking is relatively small (29 species). Why did the authors not test a larger and more diverse set of public data? This raises the question on how representative the results shown are of what users will actually encounter in their own datasets.

**Response.** We did not hand-pick the species used in SRA analysis, and tested on every species in our test data where the finished genome and raw reads were both available. Since the time of submission, new assemblies have been added to RefSeq, so we randomly selected 10 species and analyzed the raw reads. We removed one of the samples during pre-processing due to high duplication rates. We then ran RESPECT and estimated the genome length with 1X coverage SRA data with similar performance (Figure S29). We have added a note to the discussion.

**Figure S28**

6) The analysis presented in the manuscript is strictly focused on short read data (both simulated and publicly available datasets). Given the rise of long-read sequencing, it would be important to assess how their tool copes with PacBio/nanopore sequence data. At the very least, the authors should further evaluate the accuracy of RESPECT with higher error rates typical of long-read data (it seems they have only tested a 1% sequencing error rate).

**Response.** The reviewer is absolutely correct in saying that the errors in long-reads have higher error, and that RESPECT is developed for short read sequencing data. However, please note that the current practice is NOT to use long reads for *genome skimming*; instead, long-read sequencing is mostly used for high coverage sequencing followed by assembly. Once assembly is available, the motivation to run RESPECT is reduced. Importantly, even as long-read usage becomes more common, they are almost always acquired in addition to short-reads, and therefore we do not see this as a roadblock to RESPECT adaptation. The cost and properties of long reads have not motivated their use for genome skimming; when they do, RESPECT will have to be updated, but this is beyond the scope of the paper.

7) The font size of panels C and D of Figure 1 is extremely small. Suggest increasing to improve readability.

**Response.** We have increased the font size.

Reviewer #2: While I didn't have any trouble installing the dependencies it would be useful to provide a conda package or docker container prior to final publication. While not required for publication it would also be useful to test on sequencing platforms aside from Illumina, specifically if it also works on PacBio CCS data.

**Response.** We have spent a lot of effort on this, but due to RESPECT’s dependency on Gurobi solver, we have not been able to find a way to write a conda recipe when there is a dependency hosted on other channels (Gurobi channel in this case). However, as mentioned on RESPECT’s github page, the user can still use conda to easily install all dependencies. Then, they just need to download and run setup.py script to install RESPECT.

We have developed RESPECT for short read sequencing data. The reviewer is absolutely correct in saying that the errors in long-reads have higher error. However, analysing RESPECT for long-reads is beyond the scope of this paper as it will require a significant amount of tuning and training and large data-sets are not available. Importantly, even as long-read usage becomes more common, they are almost always acquired *in addition* to short-reads, and therefore we do not see this as a roadblock to RESPECT adaptation.

Reviewer #3: The paper presents an interesting technique for leveraging low-coverage sequencing data to estimate important characteristics of the underlying genome, namely the genome’s length and its k-mer repeat spectrum. The problem is well-motivated as the k-mer repeat spectrum is an informative statistic that can yield, for example, the genomic diversity across individuals in a population, and the ability to determine the spectrum with a low-computational cost makes it an attractive alternative to computationally expensive methods such as complete genome assembly. The presented approach is shown to improve on previous approaches to this problem.

An additional contribution of the paper is in providing both theoretical and empirical justification for why an initial optimization approach fails to accurately estimate the k-mer repeat spectrum. The paper points out that the initial approach fails because its estimate of the spectrum is sensitive to small differences in the initial sequencing data. The novel optimization approach introduced in the paper seeks to address this difficulty by imposing constraints on the estimate of the spectrum.

The paper is well-written, and the presented method works well even at < 1 coverage. As such, I recommend accepting the paper.

**Response.** We thank the reviewer for their comments.
